# TTP protects against acute liver failure by regulating CCL2 and CCL5 through m^6^A RNA methylation

**DOI:** 10.1172/jci.insight.149276

**Published:** 2021-12-08

**Authors:** Pingping Xiao, Mingxuan Li, Mengsi Zhou, Xuejun Zhao, Cheng Wang, Jinglin Qiu, Qian Fang, Hong Jiang, Huifen Dong, Rui Zhou

**Affiliations:** 1 Hubei Province Key Laboratory of Allergy and Immunology and; 2 Department of Parasitology, School of Basic Medical Sciences, Wuhan University, Wuhan, China.; 3 School of Basic Medical Sciences, Hubei University of Medicine, Shiyan, China.; 4 School of Pharmacy, Hubei University of Science and Technology, Xianning, China.

**Keywords:** Hepatology, Inflammation, Chemokines, Hepatitis

## Abstract

Tristetraprolin (TTP), an important immunosuppressive protein regulating mRNA decay through recognition of the AU-rich elements (AREs) within the 3′-UTRs of mRNAs, participates in the pathogenesis of liver diseases. However, whether TTP regulates mRNA stability through other mechanisms remains poorly understood. Here, we report that TTP was upregulated in acute liver failure (ALF), resulting in decreased mRNA stabilities of CCL2 and CCL5 through promotion of N^6^-methyladenosine (m^6^A) mRNA methylation. Overexpression of TTP could markedly ameliorate hepatic injury in vivo. TTP regulated the mRNA stabilization of CCL2 and CCL5. Interestingly, increased m^6^A methylation in CCL2 and CCL5 mRNAs promoted TTP-mediated RNA destabilization. Moreover, induction of TTP upregulated expression levels of WT1 associated protein, methyltransferase like 14, and YT521-B homology N^6^-methyladenosine RNA binding protein 2, which encode enzymes regulating m^6^A methylation, resulting in a global increase of m^6^A methylation and amelioration of liver injury due to enhanced degradation of CCL2 and CCL5. These findings suggest a potentially novel mechanism by which TTP modulates mRNA stabilities of CCL2 and CCL5 through m^6^A RNA methylation, which is involved in the pathogenesis of ALF.

## Introduction

Acute liver failure (ALF) is a critical disorder with a frequently fatal outcome and a 30% mortality (1). ALF has emerged as a public health issue around the world that occurs generally in patients who do not have preexisting liver disease. ALF can be caused by a series of risk factors, including paracetamol toxicity, hepatic ischemia, viral and autoimmune hepatitis, drug-induced liver injury from prescription drugs, and herbal and dietary supplements (2). Over the last decade, clinically defined overdose of acetaminophen (APAP, a dose-dependent hepatotoxin) has been the most common cause of adult ALF in the Western world, accounting for close to 50% of all cases (3). Unfortunately, there is no definitive cure for progressive ALF, which can progress to liver fibrosis, cirrhosis, hepatocarcinoma, and even death. During liver diseases, various cells, such as hepatocytes, macrophages, and endothelial cells, secrete cytokines and chemokines (CC motif chemokine ligand 2 [CCL2], CC motif chemokine ligand 5 [CCL5], CXC motif chemokine ligand 10 [CXCL10], and so on) to recruit hepatic immune cells, especially macrophages, to the injury sites (4, 5). Increasing evidence suggests animal models lacking CCL2 and CCL5 display lower immune cell activation and reduced liver injury (6, 7). Moreover, hepatocytic CCL2 has been reported to promote macrophage infiltration in APAP-induced liver injury (8).

The coordinated expression of inflammatory cytokines/chemokines involves multiple steps that determine rates of gene transcription, translation, and mRNA decay. Although transcription is the first step in the regulation of inflammatory cytokine/chemokine expression, posttranscriptional regulation, including mRNA decay, is key to control protein synthesis. The AU-rich elements (AREs) of 3′-untranslated regions (3′-UTRs) of mRNAs represent an important element in the posttranscriptional regulation of inflammatory cytokines/chemokines, which are recognized by selected members of a group of trans-acting factors of RNA-binding proteins (RBPs) (9). Tristetraprolin (TTP, also known as ZFP36) is known as an RBP that recognizes the AREs within the 3′-UTRs of mRNAs and controls their stabilities in the cytoplasm (9). It is an important immunosuppressive protein because it regulates expression of several inflammatory cytokines/chemokines, including interleukin 6 (IL-6), interleukin 8 (IL-8), and CCL2, which have been reported in the pathogenesis of rheumatoid arthritis, psoriasis, and multiple sclerosis (10, 11). However, the pathological functions of TTP in liver diseases are still conflicting. TTP has been reported to increase apoptosis and inhibit activation and invasion of hepatic stellate cells in vitro (12). A recent study indicates that TTP promotes hepatic steatosis, inflammation, and fibrosis in mice fed with a methionine/choline-deficient diet (13). The role of TTP in the development of hepatic disorders remains unclear and needs further investigations.

Modifications of RNA have recently emerged as an additional regulatory layer of gene expression (14). N^6^-methyladenosine (m^6^A) is the most abundant internal modification in mammalian mRNA (15). m^6^A, as a novel epitranscriptomic marker, plays an important role in different aspects of RNA metabolism, including mRNA stability (14). The modification of m^6^A is catalyzed by methyltransferase complex that contains methyltransferase like 3 (METTL3), methyltransferase like 14 (METTL14), and WT1 associated protein (WTAP). In turn, alkB homolog 5 and alphaketoglutarate dependent dioxygenase (FTO) function as 2 mammalian RNA demethylases to reverse the m^6^A modifications (16). Members of the YT521-B homology (YTH) domain family of proteins, including YTHDF1, YTHDF2, and YTHDF3, have been identified as direct m^6^A readers. It was reported that YTHDF2 and YTHDF3 promote decay of target mRNAs (17). The physiological roles of m^6^A modification have been identified in various biological processes, including immune responses (14). Given the important role for TTP in immune responses by regulating mRNA stability (12), it is possible that TTP may cross-act with m^6^A-mediated RNA degradation to regulate the inflammatory responses in the liver.

In this report, we investigated the potentially novel function and specific mechanism of TTP in alleviating ALF induced by CCL4 or APAP stimulation. Our findings indicate that TTP deficiency leads to a global decrease in m^6^A RNA methylation through regulation of WTAP, METTL14, and YTHDF2. Furthermore, TTP modulates mRNA stabilities of CCL2 and CCL5 by regulating RNA m^6^A methylation, contributing to its role in ALF. Therefore, our findings illustrate that TTP attenuates the expression of CCL2 and CCL5 in an m^6^A modification–dependent manner and serves as a potentially new molecule to alleviate ALF, which may provide a useful strategy for treating liver dysfunction.

## Results

### TTP protects against ALF in APAP- or CCl4-treated mice.

**Figure 1:**
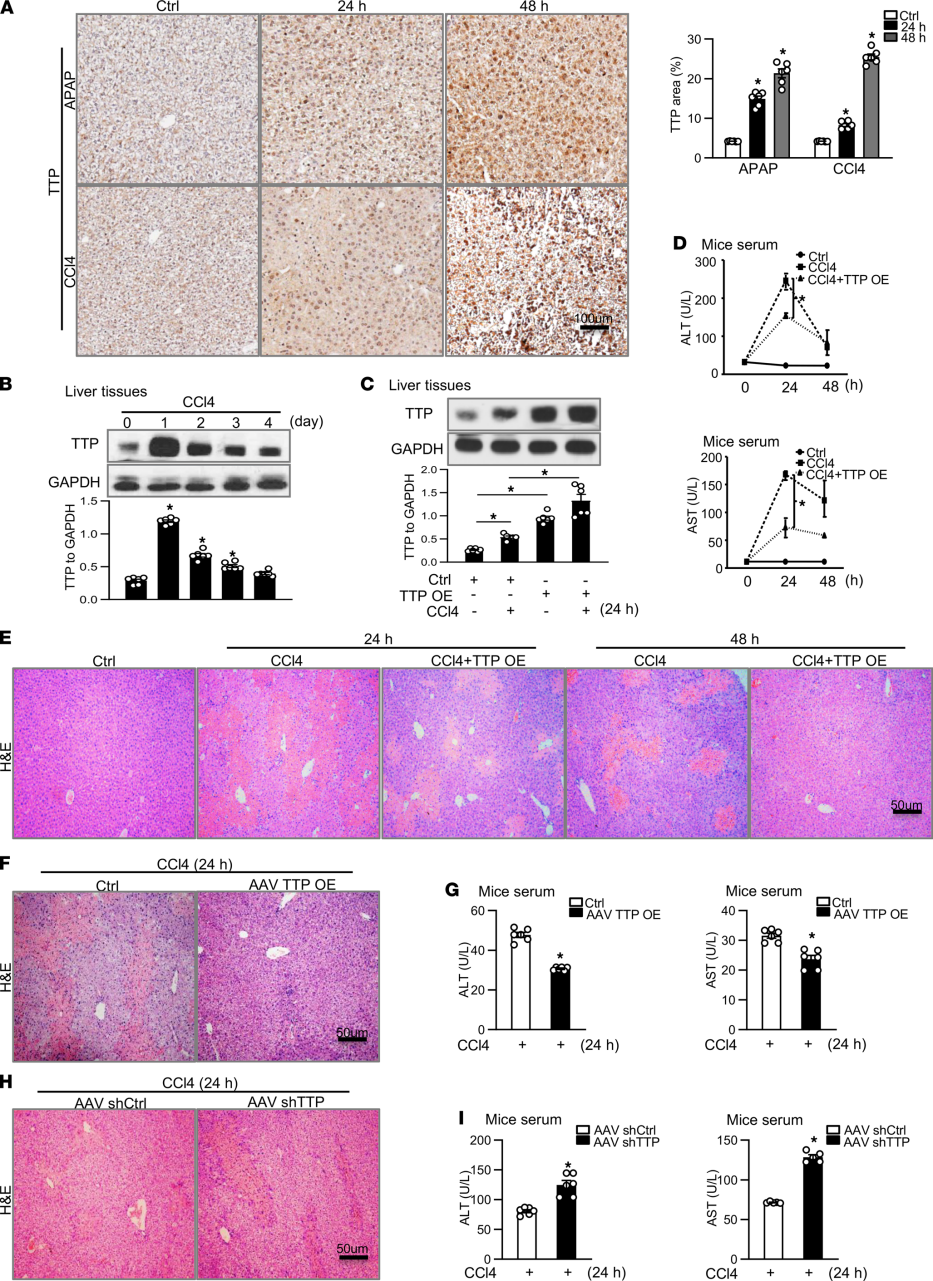
TTP protects against acute liver failure in APAP- or CCl4-treated mice. (**A**) Expression of TTP protein was detected in the liver from APAP- or CCl4-induced mice for different times by immunohistochemistry (scale bar = 100 μm). Graph shows TTP protein levels ± SEM (*n* = 6). (**B**) Expression of TTP protein was detected in CCl4-challenged mice for different times with Western blot. Graph shows TTP protein levels ± SEM (*n* = 6). (**C**) Expression of TTP protein was detected in TTP-overexpression group following CCl4 injection by Western blot. Graph shows TTP protein levels ± SEM (*n* = 6). (**D**) The plasma ALT and AST level in mice (*n* = 6). (**E**) H&E staining of liver sections from TTP-overexpression mice induced by CCl4 for 24 and 48 hours. (Scale bar = 50 μm) (*n* = 6). (**F**) H&E staining of liver sections from AAV8-TTP–infected mice induced by CCl4 (scale bar = 50 μm) (*n* = 5). (**G**) The plasma ALT and AST level in mice (*n* = 6). (**H**) H&E staining of liver sections from TTP-knockdown mice induced by CCl4 (scale bar = 50 μm) (*n* = 5). (**I**) The plasma ALT and AST level in mice (*n* = 5). Data represent mean ± SEM from 3 independent experiments. Statistics by 1-way ANOVA with Dunnett’s multiple comparisons test (**A** and **B**), 2-way ANOVA with Tukey’s multiple comparisons test (**C**), 2-way repeated measure ANOVA with Tukey’s multiple comparisons test (**D**), and 2-tailed Student’s *t* test (**G** and **I**). *P < 0.05 versus Ctrl.

We initially examined whether TTP is indeed important in the pathogenesis of ALF. In the model of ALF administrated with APAP or CCl4 for 24 and 48 hours, hepatic TTP deposition was highly expressed not only in the cell cytoplasm but also in the nuclei, as shown with immunohistochemical staining (Figure 1A). The protein and mRNA expression of TTP showed a significant increase in mice treated with APAP or CCl4 compared with the controls (Figure 1, A and B; and Supplemental Figure 1, A and B; supplemental material available online with this article; https://doi.org/10.1172/jci.insight.149276DS1). The protein level of TTP reached a peak level at 24 hours in mice injected with APAP or CCl4, as shown with Western blot (Figure 1B and Supplemental Figure 1B).

To investigate the role of TTP in APAP- or CCl4-induced ALF in vivo, mice were treated with CCl4 or APAP for 24 and 48 hours, followed by hydrodynamic tail vein injection with TTP-expressing vectors or control for additional 24 hours (Supplemental Figure 1C). As expected, ectopic TTP expression was confirmed in the TTP vector–treated group compared with that in the control group (Figure 1C and Supplemental Figure 1, D–G). The overexpression of TTP in the liver was mainly concentrated in hepatocytes, and stable expression of TTP was observed in liver following TTP vector injection (Supplemental Figure 1, F and G). Notably, both alanine aminotransferase (ALT) and aspartate aminotransferase (AST) levels in TTP vector–treated mice were markedly reduced compared with those in the controls after CCl4 or APAP treatment for 24 or 48 hours (Figure 1D and Supplemental Figure 1H). H&E staining showed that the area of hepatic necrosis in the control was diminished in the TTP vector–treated group injected with CCl4 or APAP (Figure 1E and Supplemental Figure 1I). Moreover, adeno-associated virus 8 (AAV8) vectors were used to deliver TTP to mice with CCl4-induced ALF (Supplemental Figure 1J). We observed a significant increase in the hepatic expression of TTP following AAV8-TTP injection (Supplemental Figure 1, K and L) and decreased hepatic necrosis in AAV8-TTP–infected mice following CCl4 injection (Figure 1F). ALT and AST levels in AAV8-TTP–infected mice were markedly reduced compared with those in the controls after CCl4 treatment (Figure 1G). AAV vector expressing a short hairpin RNA (shRNA) directed at TTP (AAV-shTTP) and a control hairpin (AAV-shCtrl) were constructed to explore the cascade of TTP knockdown in ALF. The expression level of TTP was remarkably reduced in the AAV-shTTP–treated group compared with that in controls (Supplemental Figure 1, M and N). H&E staining indicated that the area of hepatic necrosis was significantly expanded in the TTP deficiency group following CCl4 treatment (Figure 1H). ALT and AST levels were significantly upregulated in TTP-knockdown mice after CCl4 injection (Figure 1I). Taken together, our data suggest that TTP expression is markedly increased in murine ALF models, and overexpression of TTP attenuates the development of APAP- or CCl4-induced ALF.

### TTP regulates CCL2 and CCL5 expression in vitro and in vivo.

**Figure 2:**
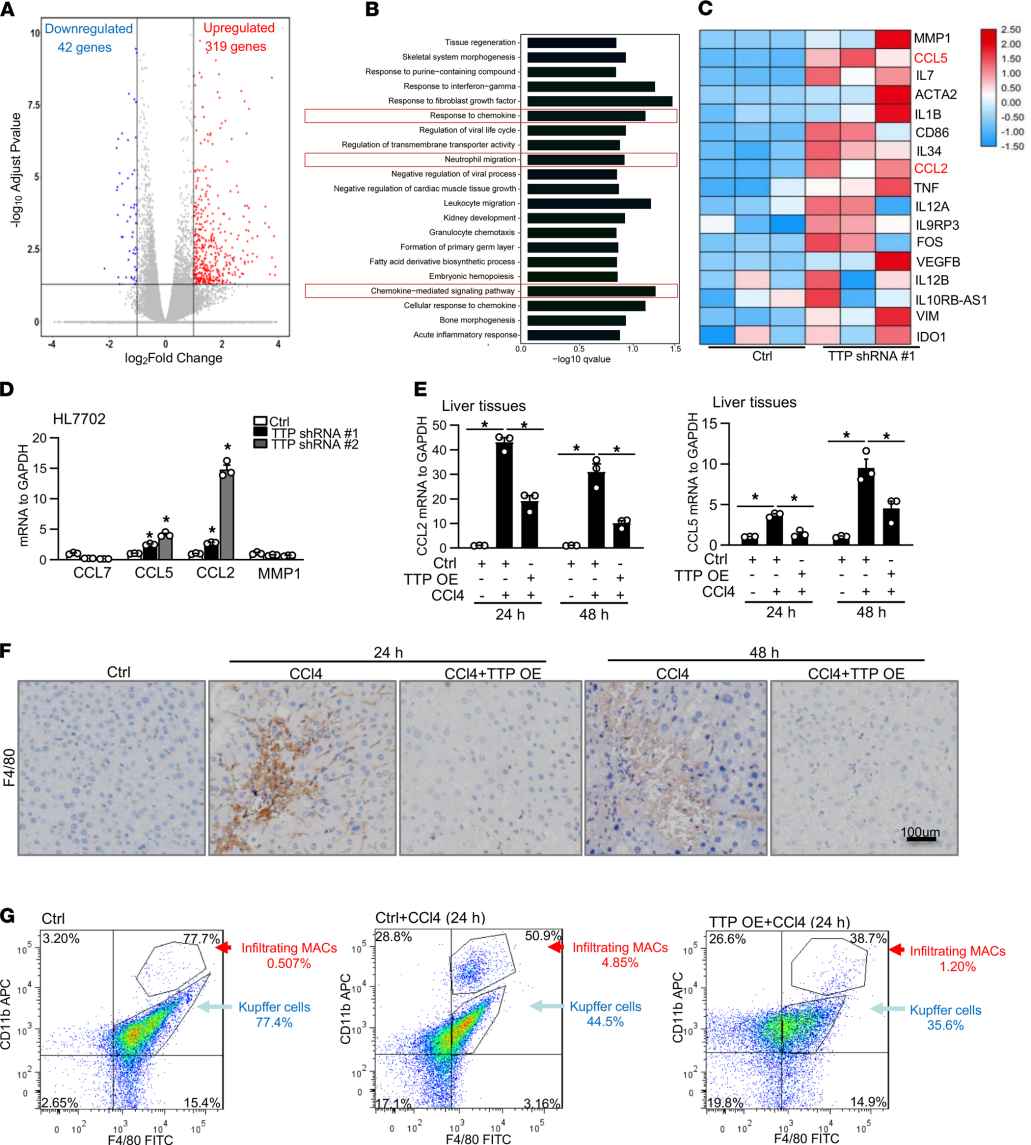
TTP regulates CCL2 and CCL5 expression in vitro and in vivo. (**A**) Volcano plots showing upregulated (red) or downregulated (blue) genes in TTP-knockdown HL7702 cells versus Ctrl, as assessed by RNA-Seq. (**B**) Gene ontology (GO) based on RNA-Seq results showing upregulated genes in TTP-knockdown cells. (**C**) Heatmap shows inflammation-related gene expression in TTP-knockdown cells. (**D**) Inflammation-related genes were measured in TTP-knockdown cells by real-time PCR. (**E**) The mRNA level of CCL2 and CCL5 in liver tissues from mice was measured by real-time PCR. (**F**) Liver sections were stained by immunohistochemistry for macrophage surface marker F4/80 to identify and quantify hepatic macrophages (scale bar = 100 μm). Graph shows F4/80 levels ± SEM (*n* = 6). (**G**) Flow cytometry was used to quantify total liver macrophages (F4/80^+^CD11b^+^), resident Kupffer cells (F4/80^hi^CD11b^lo^), as well as infiltrating liver macrophages (F4/80^lo^CD11b^hi^) (*n* = 6). Data represent mean ± SEM from 3 independent experiments. Statistics by 1-way ANOVA with Dunnett’s multiple comparisons test (**D**) and 2-way ANOVA with Tukey’s multiple comparisons test (**E**). *P < 0.05 versus Ctrl.

To clarify the potential role of TTP in protecting against ALF, we designed 2 shRNAs (Supplemental Table 1) to target different regions of TTP to knock down TTP expression and used a plasmid to overexpress TTP (Phage-TTP). The efficiency of TTP shRNAs and phage-TTP was confirmed in HL7702 cells (Supplemental Figure 2, A and B). We then investigated the effects of loss of function of TTP on gene expression by using RNA-Seq. RNA-Seq analysis showed that a total of 361 genes (fold change ≥ 0.5 log_2_ and *P* ≤ 0.05) were changed in TTP-knockdown cells (Figure 2A). Among them, 319 genes were upregulated and 42 genes were downregulated by TTP knockdown, as shown with volcano plot and pie chart (Figure 2A and Supplemental Figure 2C). Gene ontology (GO) analysis revealed that genes that TTP negatively regulated were highly enriched in chemokine-related inflammatory processes, such as response to chemokines, neutrophil migration, and chemokine-mediated signaling pathways (Figure 2B). Several liver injury–related cytokines/chemokines, including CCL2, CCL5, IL-6, and IL-1β, were increased in TTP-knockdown cells (Figure 2C). Considering the important role of CCL2 and CCL5 in ALF (6, 7), we then chose CCL2 and CCL5 for further study. Upregulation of CCL2 and CCL5 in TTP-knockdown HL7702 cells was shown by using real-time PCR (Figure 2D). The expression of CCL2 and CCL5 was also upregulated in U937 cells transfected with TTP shRNA (Supplemental Figure 2D). Overexpression of TTP significantly decreased the expression of CCL2 and CCL5 at the mRNA level in HL7702 cells (Supplemental Figure 2E).

To further confirm TTP regulates CCL2 and CCL5 expression in vivo, we isolated primary hepatocytes and macrophages from mice following CCl4 treatment. A significant increase of CCL2 and CCL5 was detected in primary hepatocytes and macrophages from mice treated with CCl4 (Supplemental Figure 2F). Moreover, the expression of CCL2 and CCL5 was measured in CCl4- or APAP-treated mice injected with TTP vectors or AAV-shTTP. Consistent with in vitro data, a significant decrease of CCL2 and CCL5 was detected in the liver tissues from mice treated with TTP vectors, compared with those of control after injection with CCl4 or APAP for 24 and 48 hours (Figure 2E and Supplemental Figure 2, G–I). Also, the expression of CCL2 and CCL5 was significantly increased in TTP-knockdown mice (Supplemental Figure 2J). Furthermore, we detected significantly reduced expression levels of IL-6 and IL-1β in TTP-overexpressed mice following CCl4 treatment (Supplemental Figure 2K). The expression of IL-6 and IL-1β was remarkably increased in TTP-knockdown mice after CCl4 injection (Supplemental Figure 2K).

CCL2 and CCL5 exhibit a strong chemotactic activity for monocytes, which plays an important role in ALF (18). To clarify the role of TTP in inflammatory cell infiltration, we tested CCL2- or CCL5-induced chemotaxis activity associated with TTP. As previously reported (18), we chose U937 cells to coculture with HL7702 cells. The knockdown of TTP increased U937 cell migration compared with control (Supplemental Figure 2L). TTP overexpression showed a significant inhibitory effect on U937 cell migration (Supplemental Figure 2L). Moreover, we detected the expression of F4/80 in TTP vector–treated mice injected with CCl4 or APAP by using immunohistochemistry. As expected, the expression of F4/80 (a macrophage marker) was significantly increased in the mice following APAP or CCl4 injection for 24 and 48 hours compared with the controls (Figure 2F and Supplemental Figure 2M). Importantly, a marked reduction in F4/80 expression was detected in TTP vector–treated mice following CCl4 or APAP treatment (Figure 2F and Supplemental Figure 2, M and N). Furthermore, F4/80 was significantly elevated in TTP deficiency mice after CCl4 or APAP injection (Supplemental Figure 2O). To examine the state of resident and infiltrating macrophages in the TTP-overexpressed group following CCl4 treatment, we analyzed the F4/80^+^CD11b^+^ population of liver mononuclear cells with flow cytometry. CCl4 treatment induced a marked increase in the number of F4/80^lo^CD11b^hi^ infiltrating macrophages (Figure 2G). Notably, a remarkable decrease of F4/80^lo^CD11b^hi^ infiltrating macrophages was shown in the TTP-overexpression group following CCl4 treatment (Figure 2G). A significant increase in the number of F4/80^lo^CD11b^hi^ infiltrating macrophages was observed in TTP-knockdown mice after CCl4 injection (Supplemental Figure 2P). These results collectively suggest that TTP negatively regulates CCL2 and CCL5 expression and infiltration of circulating mononuclear cells in ALF.

### TTP mediates m^6^A RNA methylation level by regulating WTAP, METTL14, and YTHDF2.

**Figure 3:**
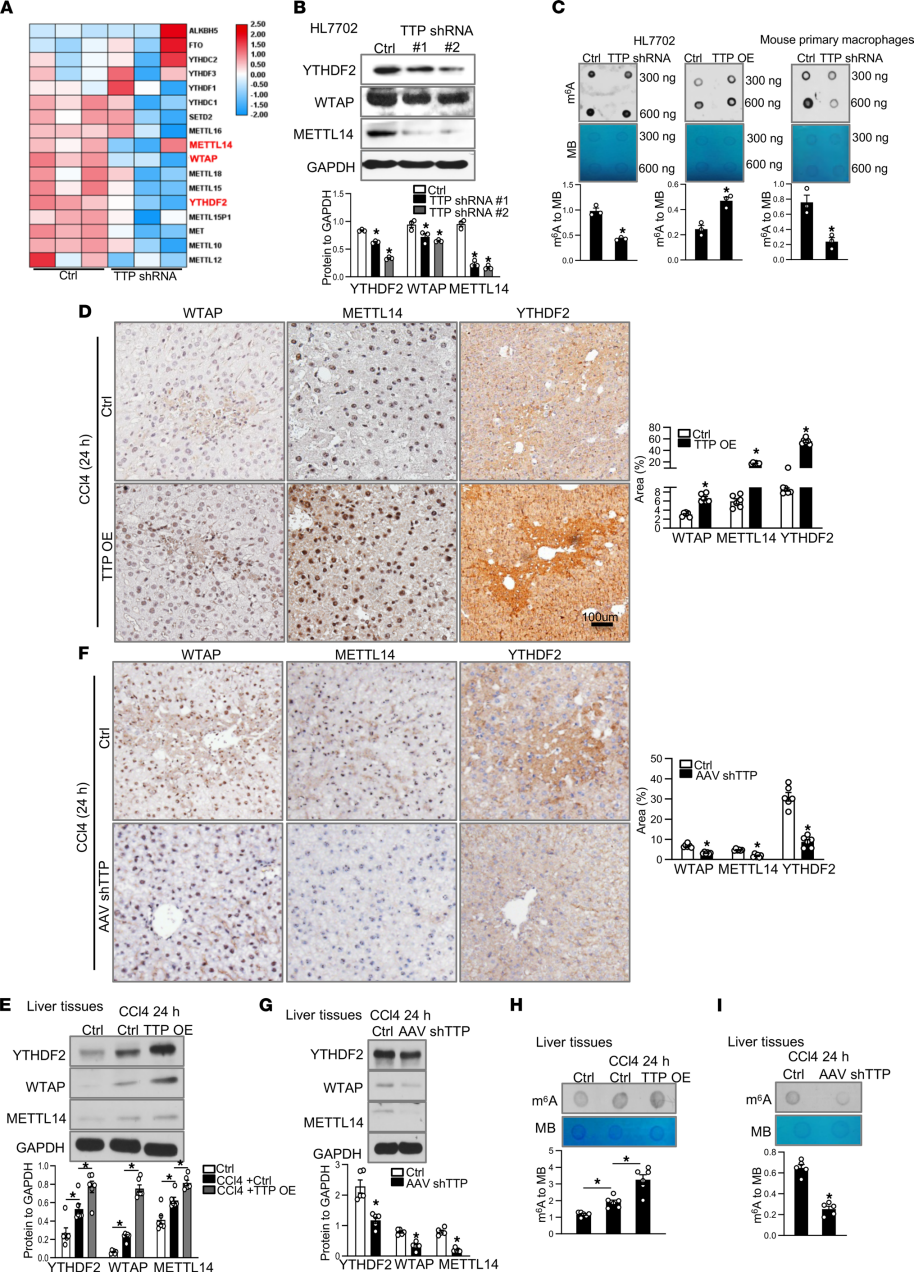
TTP mediates m^6^A RNA methylation level by regulating WTAP, METTL14, and YTHDF2. (**A**) Heatmap shows m^6^A-associated gene expression in TTP-knockdown cells. (**B**) Western blot for WTAP, METTL14, and YTHDF2 protein in TTP-knockdown cells. Graph shows protein levels ± SEM. (**C**) Dot blot was used to measure total m^6^A levels in TTP-knockdown or -overexpression cells. (**D** and **E**) Expression of WTAP, METTL14, and YTHDF2 protein was detected in injured liver after TTP-overexpression vector injection for 24 hours with immunohistochemistry (scale bar = 100 μm) and Western blot. Graph shows protein levels ± SEM (*n* = 6). (**F** and **G**) Expression of WTAP, METTL14, and YTHDF2 protein was detected in TTP deficiency mice liver induced by CCl4 (scale bar = 100 μm) and Western blot. Graph shows protein levels ± SEM (*n* = 5). (**H**) The level of m^6^A modification was measured in injured liver after TTP-overexpression vector injection. Methylene blue (MB) staining acted as the loading control for the dot blot. (**I**) The level of m^6^A modification was measured in TTP-knockdown mice induced by CCl4. Data represent mean ± SEM from 3 independent experiments. Statistics by 1-way ANOVA with Dunnett’s multiple comparisons test (**B**), 2-tailed Student’s *t* test (**C**, **D**, and **F**–**I**), and 2-way ANOVA with Tukey’s multiple comparisons test (**E** and **H**). *P < 0.05 versus Ctrl.

Several zinc finger proteins have been reported to influence m^6^A modification (19). To clarify whether TTP regulates m^6^A RNA methylation, we checked the expression of key proteins involved in m^6^A methylation based on our RNA-Seq data. Interestingly, the expression levels of WTAP, METTL14, and YTHDF2 were significantly decreased in TTP-knockdown cells (Figure 3A). We further confirmed the downregulation of WTAP, METTL14, and YTHDF2 in TTP-knockdown cells by using real-time PCR (Supplemental Figure 3, A–C). Moreover, a decreased protein level of WTAP, METTL14, and YTHDF2 was observed in TTP-knockdown cells (Figure 3B and Supplemental Figure 3D). Overexpression of TTP significantly increased the expression of WTAP, METTL14, and YTHDF2 in HL7702 cells (Supplemental Figure 3, E and F).

TTP has been reported to act as a transcriptional cofactor and regulates gene expression at the transcriptional level (20). To delineate the molecular mechanism underlying the regulation of WTAP, METTL14, and YTHDF2 by TTP, we constructed luciferase reporter plasmids by cloning the WTAP, METTL14, and YTHDF2 promoters into the pGL-basic 3.0 vector. Knockdown of TTP effectively inhibited the WTAP, METTL14, and YTHDF2 promoter-luciferase reporter activities determined by luciferase reporter assay in HL7702 cells. Overexpression of TTP remarkably elevated these promoter activities (Supplemental Figure 3G). ChIP analysis revealed that there was an increased binding of TTP to the extensively characterized transcription start sites of WTAP, METTL14, and YTHDF2 in HL7702 cells (Supplemental Figure 3H). Together, these data demonstrate that TTP promotes transcription of WTAP, METTL14, and YTHDF2 through binding to their promoter regions.

Considering that TTP can positively regulate WTAP, METTL14, and YTHDF2, we speculate whether TTP has an effect on m^6^A modification. To validate this conjecture, dot blot analysis was conducted to detect the m^6^A level in TTP-knockdown cells. It showed a global decrease of m^6^A level in TTP-knockdown cells, compared with the control (Figure 3C). m^6^A level was notably augmented in TTP-overexpression cells (Figure 3C). The same result was observed in mouse primary macrophages (Figure 3C). These results were further confirmed by m^6^A RNA methylation assay in TTP-knockdown and TTP-overexpression cells (Supplemental Figure 3I).

To confirm whether TTP increases m^6^A RNA methylation level by regulating WTAP, METTL14, and YTHDF2 in vivo, the expression levels of WTAP, METTL14, and YTHDF2 were measured in mice injected with TTP-overexpression vectors or AAV-shTTP. Consistent with in vitro data, a significant increase of WTAP, METTL14, and YTHDF2 was detected in CCl4-injured livers treated with TTP-overexpression vectors (Figure 3, D and E). Lower protein levels of WTAP, METTL14, and YTHDF2 were observed in TTP-deficiency mice injected with CCl4 (Figure 3, F and G). To further assess the function of TTP on m^6^A methylation in vivo, the global m^6^A levels were measured in livers injected with TTP-overexpression vectors or AAV-shTTP by using dot blot analysis. Mice receiving TTP-overexpression vectors displayed a significant increase in the global m^6^A levels (Figure 3H). The global m^6^A levels were obviously decreased in TTP-deficiency mice (Figure 3I). Together, these data demonstrate that TTP mediates m^6^A RNA methylation level by regulating WTAP, METTL14, and YTHDF2.

### TTP regulates the stabilization of CCL2 and CCL5 mRNAs through m^6^A modification.

**Figure 4:**
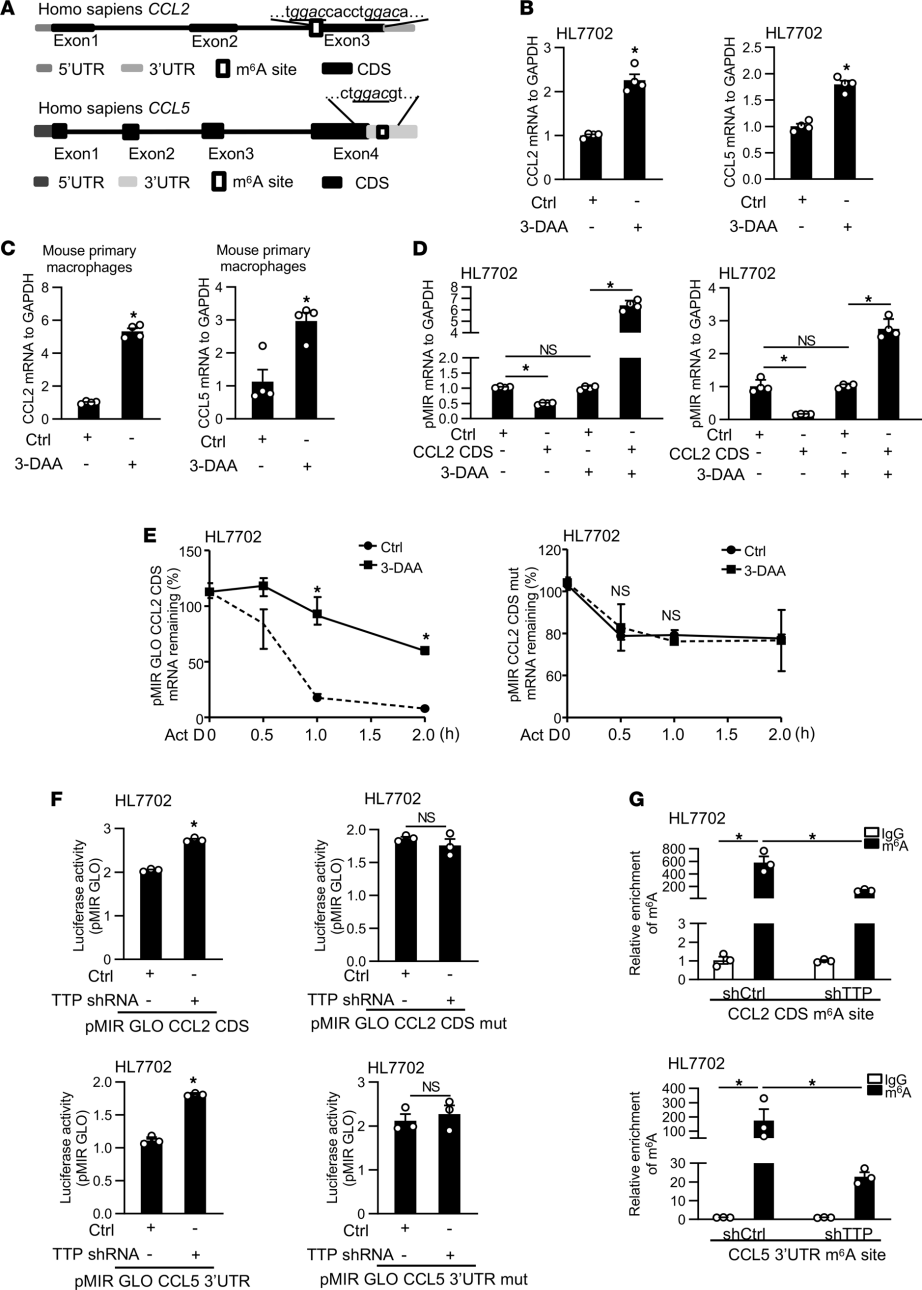
TTP regulates the stabilization of CCL2 and CCL5 mRNAs through m^6^A modification. (**A**) Schematic structures showing the potential m^6^A sequences on *Homo sapiens* *CCL2* and *CCL5*. (**B** and **C**) The mRNA level of CCL2 and CCL5 in cells treated with 3-DAA was measured by real-time PCR. (**D**) Real-time PCR analysis of luciferase levels of pMIR-GLO-CCL2-CDS or pMIR-GLO-CCL5-3′-UTR in HL7702 cells after 3-DAA treatment. (**E**) The stability of pMIR-GLO-CCL2-CDS or mutated vector mRNAs in HL7702 cells after 3-DAA treatment. (**F**) Stable TTP-knockdown cells were transfected with of pMIR-GLO-CCL2-CDS, pMIR-GLO-CCL2-CDS mut, pMIR-GLO-CCL5-3′-UTR, or pMIR-GLO-CCL5-3′-UTR mut for 48 hours, followed by luciferase analysis. (**G**) MeRIP-qPCR analysis of m^6^A enrichment on CCL2 and CCL5 in TTP-knockdown cells. Data represent mean ± SEM from 3 independent experiments. Statistics by 2-tailed Student’s *t* test (**B**, **C**, **E**, and **F**), 2-way ANOVA with Tukey’s multiple comparisons test (**D**), and 1-way ANOVA with Tukey’s multiple comparisons test (**G**). *P < 0.05 versus Ctrl. NS, no significant difference versus Ctrl.

Given the fact that TTP affects m^6^A level, we investigated whether m^6^A methylation is involved in TTP-mediated degradation of CCL2 and CCL5 mRNAs. We first analyzed the human and mouse sequences of CCL2 and CCL5 mRNAs and identified several potential m^6^A methylation sites in the sequences of CCL2 and CCL5 mRNAs (Figure 4A and Supplemental Figure 4A). To test the role of m^6^A modification in TTP-mediated CCL2 and CCL5 expression, we demonstrated HL7702 cells and mouse primary macrophages treated with 3-deazaadenosine (3-DAA), an inhibitor of global methylation, which inhibits m^6^A modification, effectively upregulated the expression of CCL2 and CCL5 (Figure 4, B and C). We then generated 2 sets of constructs containing the luciferase cDNA fused to the CCL2 coding sequences (CDSs) and CCL5 3′-UTR containing m^6^A methylation sites (pMIR-GLO-CCL2-CDS and pMIR-GLO-CCL5-3′-UTR) and the CCL2 CDS and CCL5 3′-UTR with mutation in m^6^A methylation sites (pMIR-GLO-CCL2-CDS mut and pMIR-GLO-CCL5-3′-UTR mut). HL7702 cells were transfected with pMIR-GLO-CCL2-CDS and pMIR-GLO-CCL5-3′-UTR for 24 hours in the presence or absence of 3-DAA. The luciferase mRNA of pMIR-GLO-CCL2-CDS or pMIR-GLO-CCL5-3′-UTR decreased in HL7702 cells, compared with that of controls (Figure 4D). Moreover, 3-DAA treatment resulted in a significant increase in mRNA level of pMIR-GLO-CCL2-CDS or pMIR-GLO-CCL5-3′-UTR (Figure 4D). Luciferase mRNA stability was measured in HL7702 cells transfected with these constructs in the presence or absence of 3-DAA. As shown in Figure 4E and Supplemental Figure 4B, HL7702 cells treated with 3-DAA displayed a significant increase in luciferase mRNA stability compared with the control. In cells transfected with the mutant constructs, 3-DAA treatment did not change the mRNA stability of luciferase (Figure 4E and Supplemental Figure 4B).

To determine whether m^6^A is involved in TTP regulating the expression of CCL2 and CCL5, HL7702 cells were transfected with TTP shRNA or phage-TTP (overexpression of TTP) for 48 hours, followed by luciferase analysis. TTP knockdown resulted in a significant increase in luciferase activity of pMIR-GLO-CCL2-CDS and pMIR-GLO-CCL5-3′UTR, compared with that in the control (Figure 4F). In contrast, TTP overexpression induced a decrease in luciferase activity of these constructs (Supplemental Figure 4C). In cells transfected with the mutant constructs with mutations in m^6^A methylation sites, the change of TTP did not change the luciferase activity, compared with the control (Figure 4F and Supplemental Figure 4C). We next sought to investigate whether m^6^A sites in the CDS of CCL2 mRNA and 3′-UTR of CCL5 mRNA are affected by TTP depletion by using m^6^A immunoprecipitation–quantitative PCR (MeRIP-qPCR) (the primers of CCL2 cover 2 m^6^A sites). Our data confirmed that m^6^A methylation was readily detectable on CCL2 CDS m^6^A site and CCL5 3′-UTR m^6^A site. TTP knockdown further decreased the m^6^A level of CCL2 CDS m^6^A site and CCL5 3′-UTR m^6^A site compared with the control (Figure 4G). Collectively, these results identify that TTP increases CCL2 and CCL5 mRNA degradation through promoting m^6^A modification.

### WTAP, METTL14, and YTHDF2 regulate the stabilization of CCL2 and CCL5 mRNA through targeting m^6^A methylation sites.

**Figure 5:**
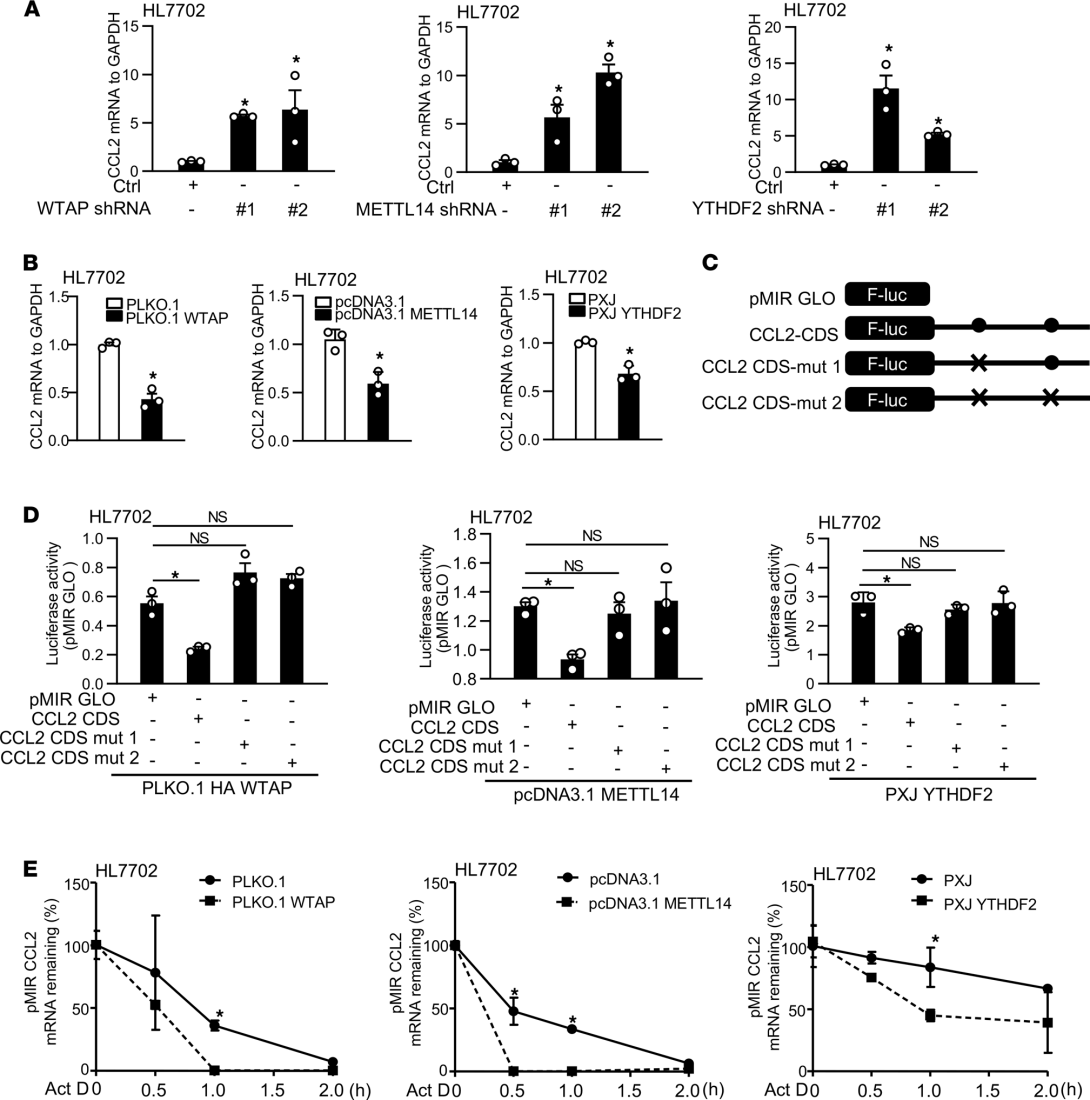
WTAP, METTL14, and YTHDF2 regulate the stabilization of CCL2 and CCL5 mRNAs through targeting m^6^A methylation sites. (**A**) The effects of WTAP, METTL14, and YTHDF2 knockdown on expression levels of CCL2 in HL7702 cells. (**B**) Effects of WTAP, METTL14, and YTHDF2 overexpression on CCL2 expression levels in HL7702 cells. (**C**) Schematic representation of mutation in m^6^A sites. (**D**) HL7702 cells were cotransfected with the luciferase construct containing the coding sequence of CCL2 (pMIR-GLO-CCL2-CDS) or CCL2 coding sequence with mutation (pMIR-GLO-CCL2-mut 1, pMIR-GLO-CCL2-mut 2) and WTAP, METTL14, and YTHDF2 overexpression plasmids for 48 hours, followed by luciferase analysis. (**E**) The stability of CCL2 mRNA was calculated in HL7702 cells transfected with WTAP, METTL14, or YTHDF2 overexpression plasmid. Data represent mean ± SEM from 3 independent experiments. Statistics by 1-way ANOVA with Dunnett’s multiple comparisons test (**A**), 2-tailed Student’s *t* test (**B** and **E**), and 1-way ANOVA with Tukey’s multiple comparisons test (**D**). *P < 0.05 versus Ctrl.

Our data indicated that TTP alters m^6^A by regulating WTAP, METTL14, and YTHDF2 expression. We then asked whether WTAP, METTL14, and YTHDF2 were involved in the regulation of CCL2 and CCL5. Several shRNAs for WTAP, METTL14, or YTHDF2 were designed to target different regions according to previous studies (21). All of them could effectively knock down the expression of WTAP, METTL14, or YTHDF2 (Supplemental Figure 5A). Knockdown of WTAP, METTL14, or YTHDF2 increased the mRNA expression levels of CCL2 and CCL5 in HL7702 cells (Figure 5A and Supplemental Figure 5B). Moreover, overexpression of WTAP, METTL14, or YTHDF2 significantly decreased the expression of CCL2 and CCL5 (Figure 5B and Supplemental Figure 5C). To clarify whether regulation of CCL2 and CCL5 is dependent on m^6^A methylation, HL7702 cells were cotransfected with plasmids that overexpress WTAP, METTL14, or YTHDF2 and constructs containing the potential m^6^A methylation sites of CCL2 or CCL5 or mutation in the m^6^A methylation sites (Figure 5C and Supplemental Figure 5D). The luciferase activity of plasmids containing m^6^A methylation sites of CCL2 or CCL5 was significantly decreased in HL7702 cells, compared with the control (Figure 5D and Supplemental Figure 5E). Overexpression of WTAP, METTL14, or YTHDF2 significantly impaired downregulation of luciferase activity when the m^6^A methylation sites were disrupted (Figure 5D and Supplemental Figure 5E).

Previous studies reported m^6^A modification affects mRNA stability (17). To clarify whether WTAP, METTL14, or YTHDF2 regulates the mRNA stability of CCL2 and CCL5, we measured mRNA stability of CCL2 and CCL5 in cells transfected with plasmids overexpressing WTAP, METTL14, or YTHDF2. As shown in Figure 5E and Supplemental Figure 5F, HL7702 cells transfected with vectors expressing WTAP, METTL14, or YTHDF2 displayed a significant decrease in CCL2 or CCL5 mRNA stability compared with the control. In addition, we detected CCL2- and CCL5-induced chemotaxis activity in cells. Knockdown of WTAP, METTL14, or YTHDF2 increased U937 cell migration (Supplemental Figure 5G). Moreover, overexpression of WTAP, METTL14, or YTHDF2 caused a significant decrease of U937 cell migration (Supplemental Figure 5H). Together, our findings suggest that WTAP, METTL14, or YTHDF2 regulates cell migration with the involvement of CCL2 and CCL5.

### TTP affects m^6^A-mediated posttranscription of CCL2 and CCL5 through regulation of WTAP, METTL14, and YTHDF2.

**Figure 6:**
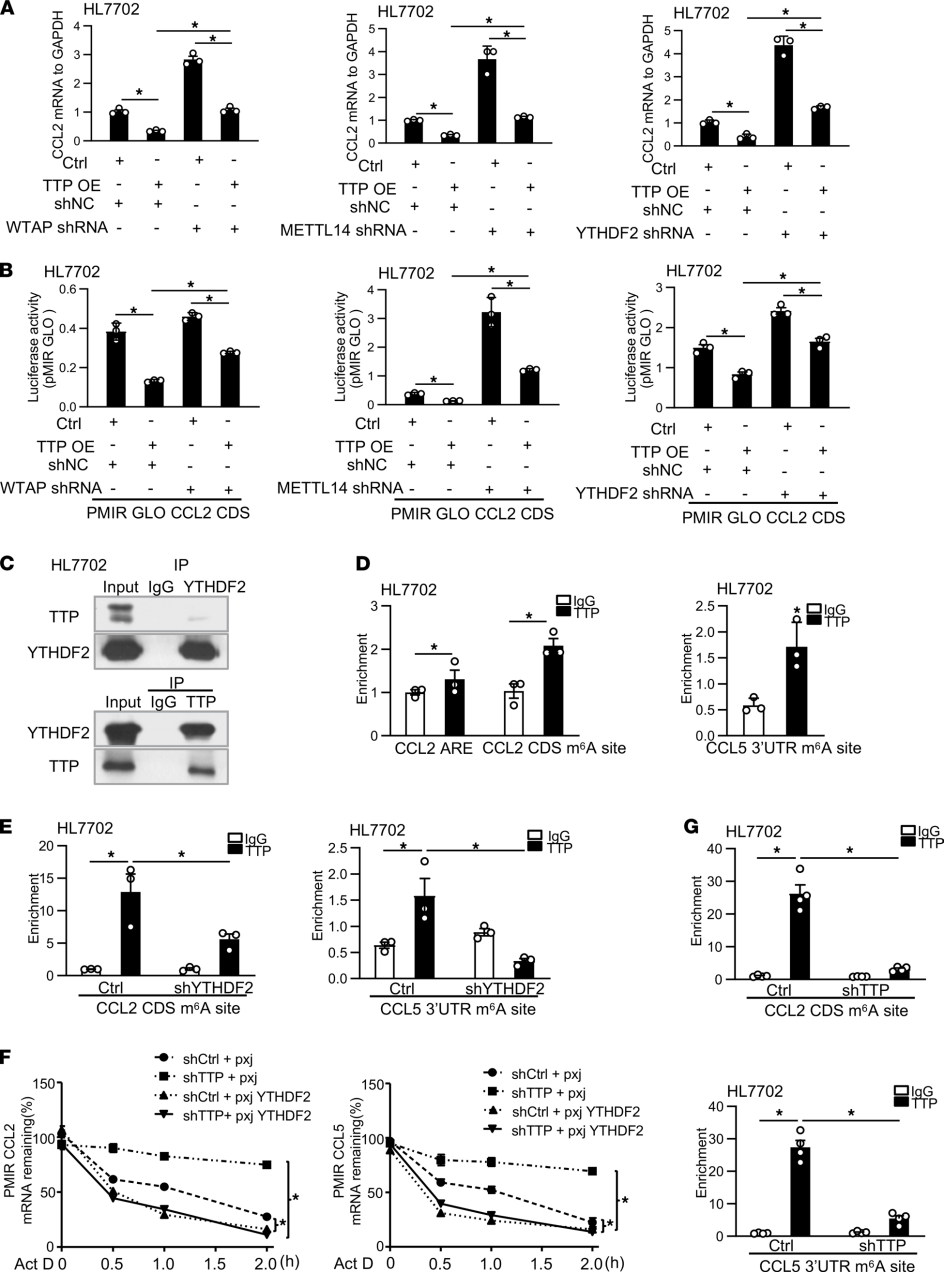
TTP affects m^6^A-mediated posttranscription of CCL2 and CCL5 through regulation of WTAP, METTL14, and YTHDF2. (**A**) The level of CCL2 was analyzed by real-time PCR in WTAP, METTL14, or YTHDF2 knockdown cells transfected with TTP-overexpression vector. (**B**) TTP-overexpression cells were cotransfected with the luciferase construct containing the CDS of CCL2 (pMIR-GLO-CCL2-CDS) and WTAP, METTL14, or YTHDF2 shRNA, followed by luciferase analysis. (**C**) The interactions between TTP and YTHDF2 were analyzed by Co-IP in HL7702 cells. (**D**) RIP was used to detect the physical association between TTP and CCL2 or CCL5 mRNAs in HL7702 cells. (**E**) RIP was used to detect the physical association between YTHDF2 and CCL2 or CCL5 mRNAs in YTHDF2-knockdown cells. (**F**) The stabilities of CCL2 and CCL5 mRNA were calculated in TTP-knockdown cells transfected with YTHDF2-overexpression plasmid. The pxj (empty vector) acted as a control for pxj YTHDF2. (**G**) iCLIP-PCR was conducted to detect TTP binding positions in m^6^A sites of CCL2 and CCL5 mRNAs in TTP-knockdown cells. Data represent mean ± SEM from 3 independent experiments. Statistics by 2-way ANOVA with Tukey’s multiple comparisons test (**A** and **B**), 2-tailed Student’s *t* test (**D**), 1-way ANOVA with Tukey’s multiple comparisons test (**E** and **G**), and 2-way repeated measures ANOVA with Tukey’s multiple comparisons test (**F**). *P < 0.05 versus Ctrl.

Given the impact of TTP on CCL2 and CCL5 mRNA stability through targeting m^6^A methylation sites and the regulation of CCL2 and CCL5 expression by several m^6^A-associated enzymes, we speculated that TTP should affect m^6^A-mediated posttranscription of CCL2 and CCL5 through regulation of WTAP, METTL14, and YTHDF2. To test this possibility, cells were transfected with Phage-TTP (overexpression of TTP) together with shRNAs of WTAP, METTL14, or YTHDF2. As shown in Figure 6A and Supplemental Figure 6A, TTP overexpression decreased the mRNA expression levels of CCL2 and CCL5 in HL7702 cells. The shRNAs of WTAP, METTL14, or YTHDF2 partially inhibited TTP-induced decrease of the expression of CCL2 and CCL5 in HL7702 cells (Figure 6A and Supplemental Figure 6A). Of note, CCL2 mRNA level was reduced in YTHDF2-depleted cells when TTP was overexpressed, probably because of the increase of ARE-mediated RNA degradation in cells overexpressing TTP. Moreover, overexpression of TTP decreased luciferase activity of PMIR-GLO-CCL2-CDS and PMIR-GLO-CCL5-3′-UTR (Figure 6B and Supplemental Figure 6B). WTAP, METTL14, or YTHDF2 shRNAs blocked the increase of luciferase activity mediated by TTP overexpression (Figure 6B and Supplemental Figure 6B). These data suggest that TTP affects CCL2 and CCL5 expression through regulation of WTAP, METTL14, and YTHDF2. In addition, to explore whether monocyte migration mediated by TTP is dependent on WTAP, METTL14, or YTHDF2, HL7702 cells with the WTAP, METTL14, or YTHDF2 knockdown were transfected with Phage-TTP for 48 hours. Overexpression of TTP caused a significant decrease of U937 cell migration (Supplemental Figure 6C). WTAP, METTL14, or YTHDF2 knockdown blocked the decrease of U937 cell migration mediated by TTP overexpression (Supplemental Figure 6C).

To further explore the molecular mechanism by which TTP promotes CCL2 and CCL5 stability through m^6^A modification, we performed immunoprecipitation experiments within HL7702 cells transfected with Phage-TTP followed by immunoblotting proteins involved in m^6^A methylation. Unexpectedly, WTAP and METTL14 were not detected by coimmunoprecipitation (Co-IP) with TTP antibody (data not shown). Strikingly, we verified a specific interaction between TTP and YTHDF2 in HL7702 cells (Figure 6C). Next, we sought to identify whether intracellular TTP interacts with m^6^A methylation sites with formaldehyde cross-linking RBP immunoprecipitation (RIP) in vivo. We detected an obvious presence of TTP highly enriched in CCL2 and CCL5 m^6^A sites by using a specific anti-TTP antibody but not the IgG (Figure 6D). The ARE sequence of CCL2 that interacts with TTP was used as a positive control (Figure 6D).

Moreover, we performed RIP to determine whether TTP binds to m^6^A sites through interacting with YTHDF2. Interestingly, YTHDF2 knockdown drastically decreased the interaction of TTP and m^6^A methylation sites in CCL2 and CCL5 mRNAs (Figure 6E). In addition, we measured mRNA stability of CCL2 and CCL5 in stable TTP-knockdown cells transfected with the YTHDF2-overexpressed plasmid. As shown in Figure 6F, TTP knockdown did not increase mRNA stability of CCL2 and CCL5 in YTHDF2-overexpressed cells. To identify the TTP binding positions associated with the m^6^A sites within CCL2 and CCL5 mRNAs, we performed individual nucleotide resolution UV cross-linking and immunoprecipitation (iCLIP) PCR using specific TTP antibodies to target endogenous TTP in HL7702 cells. A predominant binding of TTP to the m^6^A sites of CCL2 and CCL5 mRNA was identified (Figure 6G). Moreover, TTP knockdown remarkably decreased the binding of TTP protein to the m^6^A sites of CCL2 and CCL5 mRNA (Figure 6G).

### m^6^A methylation is involved in TTP-mediated protection against ALF.

**Figure 7:**
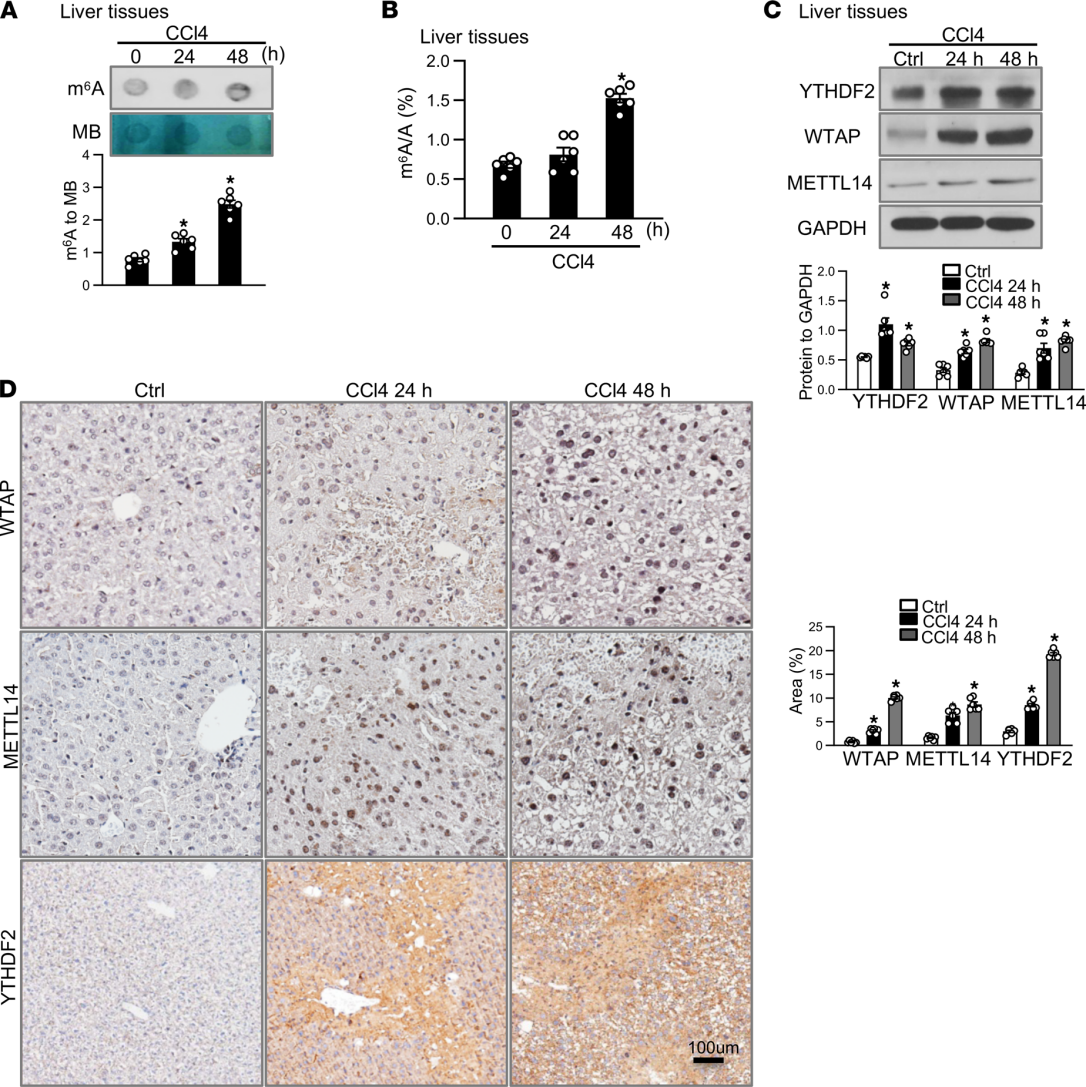
Increased expression of WTAP, METTL14, and YTHDF2 in a murine ALF model. (**A**) Dot blot was used to measure m^6^A modification in the mouse liver induced by CCl4 for different times. (**B**) The level of m^6^A modification was measured in livers induced by CCl4 for different times. (**C**) Expression of WTAP, METTL14, and YTHDF2 at the protein level was detected in the liver induced by CCl4 for 24 and 48 hours by Western blot. Graph shows protein levels ± SEM (*n* = 6). (**D**) Expression of WTAP, METTL14, and YTHDF2 protein staining was detected in livers induced by CCl4 for 24 and 48 hours by immunohistochemistry (scale bar = 100 μm). Graph shows protein levels ± SEM (*n* = 6). Data represent mean ± SEM from 3 independent experiments. Statistics by 1-way ANOVA with Dunnett’s multiple comparisons test. *P < 0.05 versus Ctrl.

Given the fact that the expression of TTP was increased in murine ALF models and TTP increased m^6^A RNA methylation level in hepatocytes and macrophages, we reasoned that m^6^A methylation might be involved in the pathogenesis of ALF. To test this possibility, the global m^6^A levels were measured in livers of mice treated with CCl4 or APAP by using dot blot analysis. The global m^6^A levels in the liver showed a significant increase in CCl4- or APAP-treated mice as compared with the control (Figure 7A and Supplemental Figure 7A). These results were confirmed by m^6^A RNA methylation assay in liver tissues from mice injected with CCl4 for 24 and 48 hours (Figure 7B). Moreover, the protein levels of WTAP, METTL14, and YTHDF2 were increased in the liver of mice treated with CCl4 or APAP for 24 and 48 hours, as shown by immunohistochemistry and Western blot (Figure 7, C and D; and Supplemental Figure 7, B and C).

**Figure 8:**
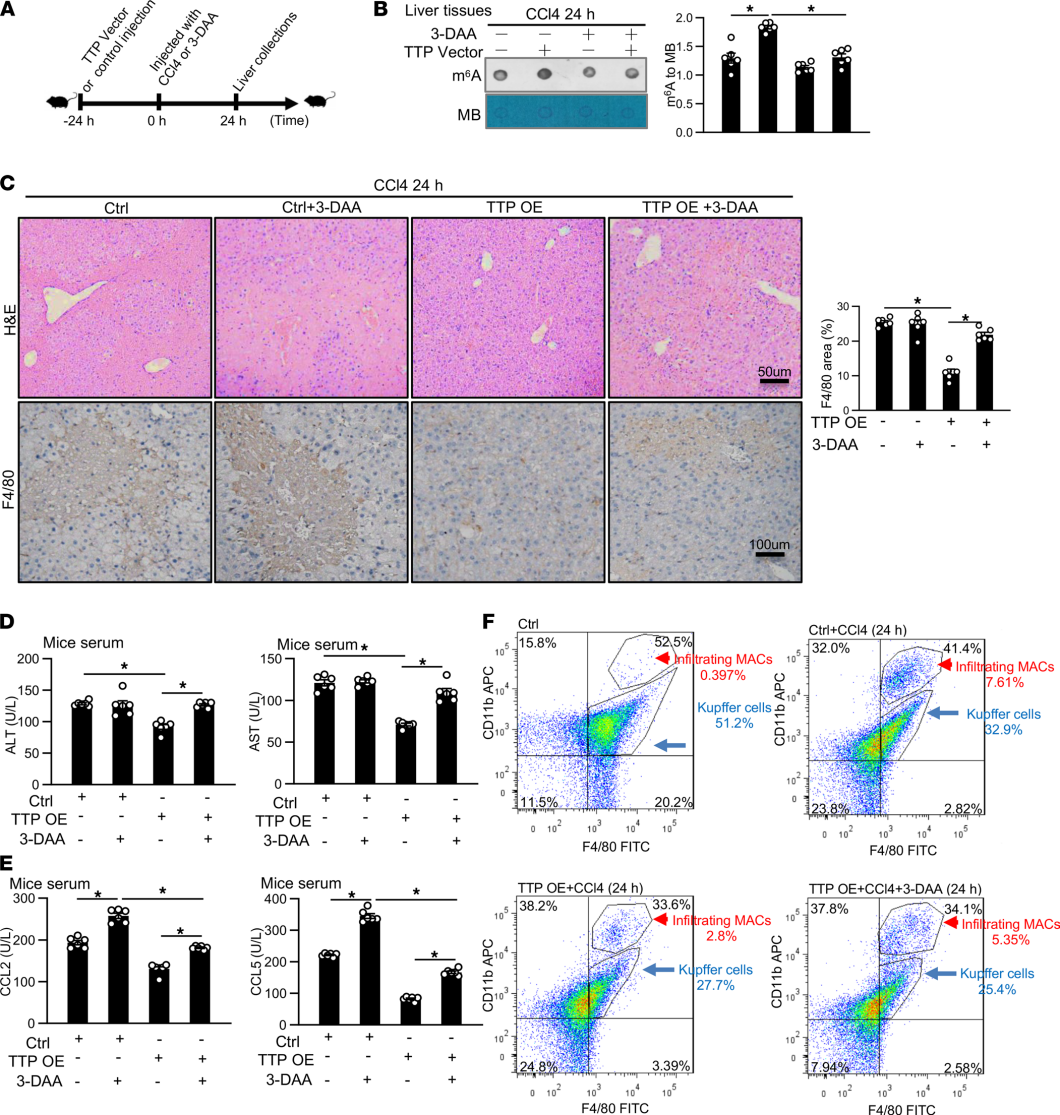
Methylation of m^6^A is involved in TTP-mediated protection against ALF. (**A**) Schematic overview of the experimental setup. Mice were first infected with TTP-overexpression vector or control for 24 hours, by hydrodynamic tail vein injection. Then, 3-DAA and/or CCl4 were injected i.p. for an additional 24 hours (*n* = 6). (**B**) Dot blot was used to measure m^6^A modification in livers induced by CCl4 after 3-DAA treatment. (**C**) H&E and F4/80 protein staining of liver sections from mice induced by CCl4 after 3-DAA treatment. (Scale bar = 50 μm or 100 μm) (*n* = 6). (**D**) The plasma ALT and AST level in mice. (**E**) Expression of CCL2 and CCL5 proteins in mice serum was measured by ELISA (*n* = 6). (**F**) Flow cytometry was used to quantify total liver macrophages (F4/80^+^CD11b^+^) and infiltrating liver macrophages (F4/80^lo^CD11b^hi^) in livers induced by CCl4 after 3-DAA treatment, as well as resident Kupffer cells (F4/80^hi^CD11b^lo^) (*n* = 6). Data represent mean ± SEM from 3 independent experiments. Statistics by 2-way ANOVA with Tukey’s multiple comparisons test. *P < 0.05 versus Ctrl.

To investigate whether TTP overexpression alleviates liver injury in mice through m^6^A methylation in vivo, 3-DAA, an inhibitor of global methylation, was used for intraperitoneal (i.p.) injection of mice. Mice were treated with CCl4 in the presence and absence 3-DAA for 24 hours, followed by hydrodynamic tail vein injection with TTP-expressing vectors for 24 hours (Figure 8A). Our dot blot data showed that 3-DAA treatment dramatically inhibited TTP overexpression–induced increase of m^6^A levels in the liver of mice injected with CCl4 for 24 hours (Figure 8B). Importantly, H&E staining revealed that 3-DAA treatment reversed the hepatic protection conferred by TTP overexpression in CCl4-challenged mice (Figure 8C). Moreover, the level of ALT and AST significantly increased in the TTP vector–treated group following 3-DAA injection in CCl4-challenged mice (Figure 8D). In addition, an increase of CCL2 and CCL5 protein level was observed in CCl4-challenged mice following 3-DAA injection (Figure 8E). Moreover, 3-DAA treatment partially blocked TTP overexpression–induced increase of CCL2 and CCL5 expression at mRNA and protein levels in CCl4-challenged mice (Figure 8E and Supplemental Figure 7D). We further examined the expression of F4/80 in livers from the CCl4-challenged mice treated with TTP vectors in the presence and absence of 3-DAA. Injection of 3-DAA significantly increased the level of F4/80 in mice with TTP overexpression following CCl4 treatment (Figure 8C). Moreover, our flow cytometry data showed that the number of F4/80^lo^CD11b^hi^ infiltrating macrophages was increased in the TTP vector–treated group injected with 3-DAA (Figure 8F). Therefore, 3-DAA treatment exaggerated liver damage in mice with TTP overexpression and nullified the protective effects of TTP.

## Discussion

The infiltration of circulating mononuclear cells (both macrophages and T cells) into the liver has been established as a feature of ALF and is associated with disease severity (22). CCL2 and CCL5 can recruit peripheral macrophages and T cells, a hallmark of ALF inflammation, and may play a central role in the pathogenesis of hepatic diseases, including ALF (23). CCL2 and CCL5 have been found to be produced by numerous cell types, including stressed hepatocytes, macrophages, and stellate cells (4, 5). Importantly, inhibition of CCL2 and CCL5 has been reported to reduce monocyte infiltration and alleviate acute liver failure (6, 7). In this study, we reveal a protective role of TTP in APAP- and CCl4-induced ALF through regulating CCL2 and CCL5 expression. Overexpression of TTP markedly decreased the infiltration of monocytes in murine ALF models. Interestingly, we demonstrate that m^6^A methylation was involved in TTP-mediated degradation of CCL2 and CCL5 mRNAs. Our findings suggest the potential for ALF treatment by targeting TTP or m^6^A status in the liver.

TTP is a well-known RBP, which recognizes and binds to AREs in the 3′-UTRs of mRNAs of multiple inflammatory cytokines and thereby induces decay or destabilization of the transcripts (9). It has been reported that TTP can act as a key regulator of the inflammatory response, especially in the fine-tuning of proinflammatory cytokines and chemokines in the liver (24). Therefore, TTP expression is tightly controlled in the cells in physiological and pathologic conditions (9). It has been reported that several signaling pathways, such as MAPK/p38 and NF-κB, are involved in regulation of TTP expression and function (25). Activation of both NF-κB and MAPK/p38 signaling pathways has been demonstrated in ALF (26) and may account for the enhanced expression of TTP in APAP- and CCl4-induced ALF.

TTP confers CCL2 mRNA instability and degradation by its interactions with the ARE region in CCL2 mRNA (27). CCL5 has been demonstrated to be negatively regulated by TTP (28). Nevertheless, there is no obvious ARE region in the 3′-UTR of CCL5 mRNA, implicating a different mechanism underlying the regulation of CCL5 by TTP. m^6^A modification modulates all stages in the life cycle of RNA, including RNA stability (15). YTHDF2 has been reported to weaken mRNA stability by recognizing and distributing m^6^A-containing mRNAs to processing bodies (29). Our study demonstrates that TTP promotes RNA stability not only through its interaction with the ARE regions within RNA transcripts but also through m^6^A-dependent mechanisms. Specifically, we found that TTP can trigger the transcription of the WTAP, METTL14, and YTHDF2 genes to promote global m^6^A methylation. We observed that TTP binds to the m^6^A sites of CCL2 mRNAs, which is consistent with a previous study (30). Moreover, TTP interacts with YTHDF2. Therefore, TTP affects mRNA fates in multiple ways. The involvement of TTP in m^6^A-mediated RNA degradation can provide a new arm of regulation by TTP for RNAs without the ARE sequences. For mRNAs containing the ARE sequences, this additional arm of regulation may play a critical role in the coordinated and dynamic expression of these genes in physiological and pathologic conditions, such as regulation of CCL2 expression in APAP- and CCL4-induced liver injury.

Recent studies suggest that some zinc finger proteins are critical regulators of m^6^A modification (19). Song et al. reported that Zfp217, functioning as a transcription activator, promotes adipogenic differentiation by mediating m^6^A demethyltransferase FTO (19). It was reported that Zc3h13 plays a critical role in anchoring WTAP, Virilizer, and Hakai in the nucleus to facilitate m^6^A methylation (31). We found that TTP triggered the transcription of the WTAP, METTL14, and YTHDF2 genes to promote global m^6^A methylation. TTP contains several tandem CCCH zinc finger domains, with the potential of interaction with DNA or RNA (9). Notably, TTP is also known as a transcriptional cofactor and can interact with several transcription factors, such as NF-κB, to regulate gene expression at the transcriptional level (20). We found that TTP can be recruited to the promoter regions of the WTAP, METTL14, and YTHDF2 gene loci and promote their transcription in hepatocytes. How TTP is recruited to these gene loci and whether it involves other transcription factors merit further investigation.

m^6^A is important for controlling many cellular and biological processes (19). Deregulation of m^6^A modification has recently been implicated in the pathogenesis of several liver-related diseases, such as nonalcoholic fatty liver disease (NAFLD), HBV, hepatitis C virus (HCV), and hepatocellular carcinoma (16). FTO was reported to promote adipogenesis in NAFLD (32). During HBV infection, m^6^A modification regulates the half-life of the HBV replication, controls the expression of HBV oncoproteins, and regulates the reverse transcriptase of pregenomic RNAs (33). In contrast, YTHDF family reader proteins were reported to inhibit HCV replication by competing for binding to Envelope to prevent virus packaging (34). However, the roles of m^6^A modification in ALF are largely unexplored. Here, we observed a marked increase of global m^6^A methylation status in ALF models. Moreover, the expression of several m^6^A-associated genes, such as WTAP, METTL14, and YTHDF2, was notably increased in APAP- and CCl4-induced ALF mice. Our data support that m^6^A modification is highly associated with the pathogenesis of ALF, suggesting the possibility of developing therapeutic strategies against ALF through targeting m^6^A modification and its related genes.

In summary, we provide compelling in vitro and in vivo evidence demonstrating that TTP protects against ALF through regulating CCL2 and CCL5 expression. Our data reveal a potentially novel mechanism of regulation of CCL2 and CCL5 by TTP through promoting m^6^A modification, suggesting potentially novel ways to protect against acute liver diseases. The insights obtained from this study further imply that transcriptional regulation of m^6^A-associated genes by TTP may represent a new mechanism of the immune-regulatory networks and is relevant to the development of new therapeutic strategies for ALF.

## Methods

### Animals and hydrodynamic tail vein injection.

Female and male BALB/c mice purchased from the Hubei Provincial Center for Disease Control and Prevention (Wuhan, China), 8 to 11 weeks old, weighing 22–25 g, were used. Female BALB/C mice were prepared for an ALF model induced by CCl4, while male BALB/C mice were prepared for an ALF model induced by APAP. Hydrodynamic injection was performed as described previously (35).

Briefly, constructed pcDNA3.1-TTP (containing complete TTP CDS) and pcDNA3.1 were suspended in saline solution and subsequently injected into the lateral tail veins of male or female mice (0.1 mL/g body weight) in fewer than 7 seconds. As for the ALF model induced by APAP or CCl4, 500 mg/kg of APAP and 1 mL/kg of CCl4 (MilliporeSigma; diluted 1 to 4 in olive oil and filtered using a 0.2 μm filter prior to administration) were i.p. injected. An m^6^A inhibitor, 3-DAA (APExBIO), was used at a final concentration of 1.5 mg/kg for mice.

### MeRIP-qPCR.

MeRIP-qPCR was performed as described previously (27). Briefly, the total RNA was extracted using TRIzol (Invitrogen, Thermo Fisher Scientific). After saving 500 ng of the total RNA as input, the remaining RNA (10 μg) was used for m^6^A-IP with m^6^A antibody (Abcam, catalog ab151230) or rabbit IgG (ABclonal, catalog AC005) in IP buffer (150 mM NaCl, 0.1% NP-40, 10 mM Tris at pH 7.4, 100 U RNase inhibitor); m^6^A RNA was immunoprecipitated with Dynabeads Protein A (Thermo Fisher Scientific) and eluted twice with 6.7 mM N^6^-methyladenosine 5′-monophosphate sodium salt at 4°C for 1 hour. The target RNA was precipitated with 5 μg glycogen, with one-tenth volume of 3 M sodium acetate in 2.5 volumes of 100% ethanol, at –80°C overnight. The m^6^A enrichment was determined by quantitative real-time PCR (qRT-PCR).

### Liver macrophage isolation and flow cytometry.

Liver macrophages were isolated from mice as described (36). Briefly, mouse livers were cut into small pieces and digested for 1 hour at 37°C in RPMI 1640 medium containing 0.05% collagenase/dispase (Roche) and 0.01% trypsin inhibitor (Gibco, Thermo Fisher Scientific). The liver suspension was pressed through a 40 μm cell strainer, then centrifuged at 800*g* for 10 minutes at 4°C. The supernatant was removed and erythrocytes were lysed with red blood cell lysis buffer for 2 minutes at 4°C, then centrifuged at 800*g* for 10 minutes at 4°C. The cell pellet was resuspended in 40% Percoll gradient (MilliporeSigma, P1644). Cell suspensions were loaded on the top of the 80% Percoll gradient and centrifuged at 376*g* for 25 minutes at room temperature without brake. The middle layer was suspended with 10 mL of RPMI 1640 medium. Cell suspensions were spun at 376*g* for 5 minutes at room temperature, and supernatant was removed. Cells were resuspended in 2% FCS and 1 × 10^6^ cells were enumerated. Subsequently, cells were stained with F4/80 (BioLegend, catalog 123107) and CD11b antibody (BioLegend, catalog 101211) for 30 minutes in the dark (0.5 μL/1 × 10^6^ cells). Finally, the cells were washed and suspended in 0.5 mL PBS. The stained cells were analyzed using flow cytometry (BD LSRFortessa). Final calculations were performed by FlowJo software (FlowJo LLC).

### Construction and stereotactic injection of vectors for TTP overexpression or knockdown.

Recombinant AAV serotype vector was a gift from Y.C. Xia (Wuhan University). Recombinant AAV vector expressing complete TTP CDS (AAV-TTP) or an shRNA directed at TTP (AAV-shTTP) was constructed and confirmed by sequencing. The recombinant plasmids were then treated as mentioned above using a triple-transfection, helper-free method and purified according to a previous study (37). Briefly, HEK293T cells (ATCC) were transfected with AAV-DJ (Addgene, catalog 130878), px552 (Addgene, catalog 60958), and AAV-helper (Addgene, catalog 81070) plasmids in 150 mm dishes at 80% confluence. The shRNA was driven by a mouse U6 promoter (pol III), and GFP was used as a reporter. The titers of virus were determined by using quantitative PCR. The final virus in PBS had a titer of 10 × 10^10^ viral particles/mL. The prepared viruses were injected by hydrodynamic tail vein injection for 14 days, then treated with 1 mL/kg of CCl4.

### Dot blotting.

Dot blots were performed using mouse monoclonal anti-m^6^A antibodies (Abcam, catalog ab208577). A total of 500–1000 ng RNA was denatured at 75°C for 5 minutes, placed on ice immediately, loaded onto Hybond N^+^ membranes, and sequentially cross-linked 5 minutes with 150 mJ/cm^2^ in the UV 254 nm Stratalinker 2400 (Analytik Jena US LLC). Membranes were blocked with 5% nonfat milk in PBS–0.1% Tween 20 at room temperature for 30 minutes and probed overnight with anti-m^6^A antibody at 4°C. HRP-conjugated secondary rabbit antibody (Proteintech, catalog HRP-60004) was incubated to spot the antigen-antibody complexes at 4°C overnight. The MB (HUSHI) staining acted as a loading control for dot blots.

### RNA-Seq.

Stable TTP-knockdown cells were used to construct libraries with the Illumina TruSeq RNA Preparation Kit according to the manufacturer’s instructions. Differential expression was determined between TTP shRNA–treated and control cells. Each group was done in 3 independent replicates. Counts were normalized with the fragments per kilobase per million values. The expression levels of transcripts were calculated using String Tie software (v1.3.4d). Other parameters were set to defaults. The RNA-Seq FASTQ files were deposited in National Center for Biotechnology Information’s Gene Expression Omnibus (GEO) and are accessible through GEO accession number GSE157581. RNA-Seq was performed by Kindstar.

### RIP-PCR.

RIP assay was performed as previously described (38). IgG was used as a negative control (Abcam, catalog ab125900). Primers used for RIP are shown in Supplemental Table 1.

### iCLIP-PCR.

iCLIP-PCR was performed as previously described (39). Briefly, TTP stably transfected cells were irradiated with 150 mJ/cm^2^ at 254 nm in a Stratalinker 2400. We scraped off the cells with cell lifters, centrifuged at 514*g* at 4°C for 1 minute to pellet cells, and then removed supernatant. We resuspended the cell pellet in 1 mL lysis buffer (with 1:100 Protease Inhibitor Cocktail Set III from Biosharp). Dynabeads Protein A was used to couple with 2–10 μg antibody per experiment for 30–60 minutes at room temperature. Then the lysates were added to the beads and rotated for 1 hour or overnight at 4°C. We placed the lysates on a magnet and discarded the supernatant, then washed twice with high-salt washing buffer (rotating the second wash for at least 1 minute at 4°C). RNA 3′ end was dephosphorylated with PNK buffer (with 5× PNK pH 6.5 buffer, PNK, RNasin). We loaded the samples on an SDS-PAGE gel and transferred the protein–RNA complexes from the gel to a Protran Nitrocellulose Membrane (MilliporeSigma). We isolated the protein–RNA complexes with phenol/chloroform and reversed protein–RNA complexes with a cDNA reverse transcription kit (TaKaRa). The enrichment was determined by qRT-PCR.

### Availability.

The RNA-Seq FASTQ files were deposited in GEO (https://www.ncbi.nlm.nih.gov/geo/query/acc.cgi?acc=GSE157581). The GEO accession number is GSE157581.

### Statistics.

All data are presented as means ± SEM of 3 experiments. Analysis was performed using 2-tailed Student’s *t* test or 1- or 2-way ANOVA test when appropriate. *P* values less than 0.05 were considered significant.

### Study approval.

All animal experiments were performed in accordance with and with the approval of the Animal Care and Use Committee of Wuhan University guidelines and regulations of Wuhan University.

## Author contributions

PX, ML, and RZ conceived the project. PX, ML, MZ, JQ, and CW designed and performed the experiments. PX, ML, QF, and XZ performed the animal experiments. PX, ML, HJ, and HD performed the statistical analysis. PX, ML, and RZ drafted the manuscript. All authors discussed the results and approved the final manuscript.

## Supplementary Material

Supplemental data

## Version 1 12/08/2021

Electronic publication
