# Supplementary material for: TTP protects against acute liver failure by regulating CCL2 and CCL5 through m6A RNA methylation
Source: JCI Insight. 2021 Dec 8;6(23):e149276. doi: 10.1172/jci.insight.149276 (PMC8675193; doi:10.1172/jci.insight.149276)
Supplement: Supplemental data [file jciinsight-6-149276-s028.pdf]

Supplemental Figure S1

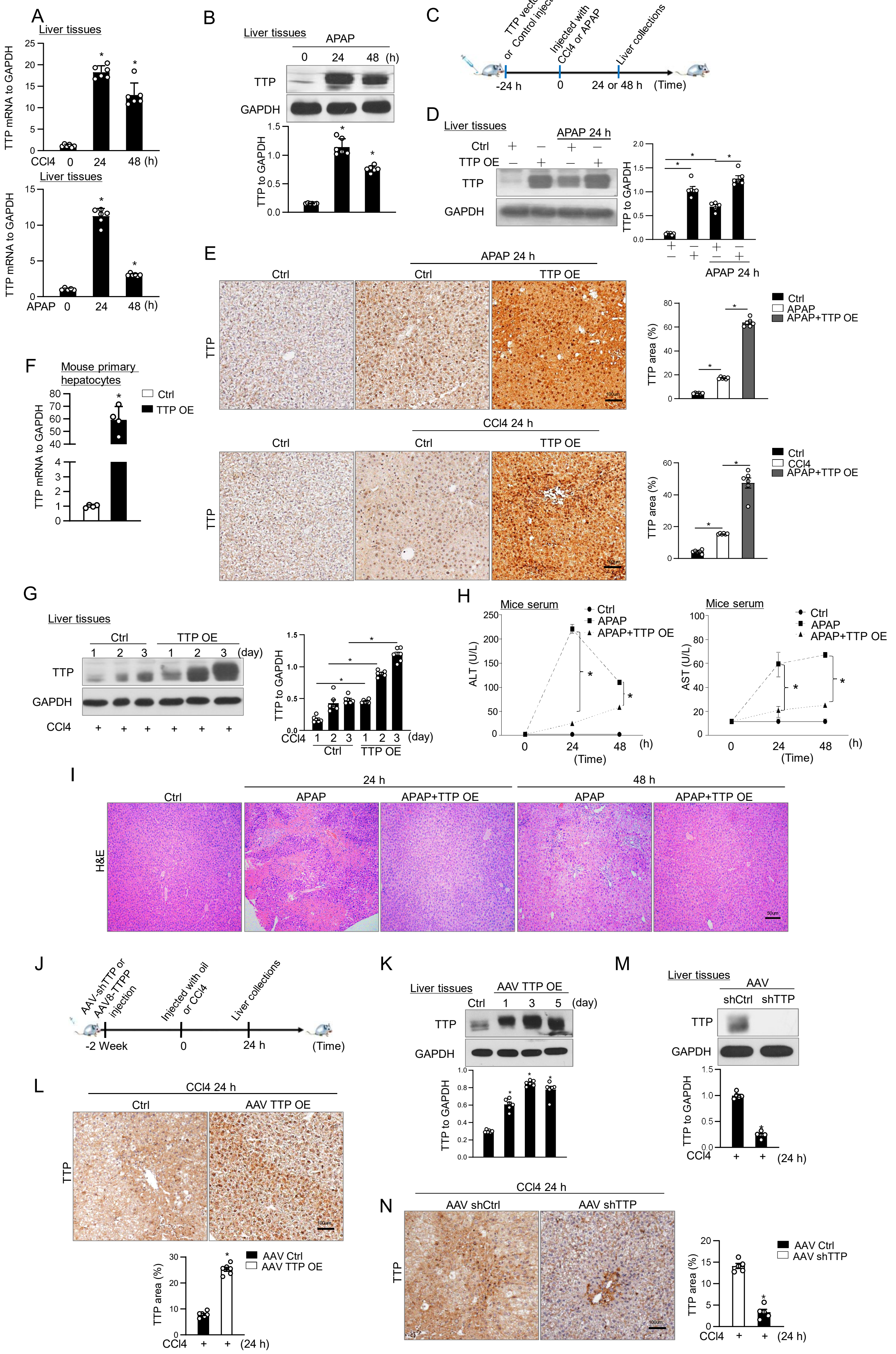

Figure S1. TTP protects against ALF in APAP- or CCl4-treated mice. (A) Expression of TTP mRNA was measured in mice livers by real-time PCR. (B) Expression of TTP protein was detected in APAP challenged mice for different time. Graph shows TTP protein levels ± SEM (n=6). (C) Schematic overview of the experimental setup. Mice were first infected with TTP vectors or controls for 24 h by hydrodynamic tail-vein injection. Then, mice were injected CCl4 or APAP intraperitoneally for additional 24 and 48 h. (D-E) Expression of TTP protein was detected in TTP overexpression group following CCl4 and APAP injection by using Western blot and immunohistochemistry. Graph shows TTP protein levels ± SEM (n=6). (F) Expression of TTP mRNA was measured in hepatocytes by real-time PCR. (G) Expression of TTP protein was measured in mice liver by Western blot (n=6). (H) The plasma ALT and AST level in mice. Graph shows TTP protein levels ± SEM (n=6). (I) H&E staining of liver sections from mice induced APAP for 24 and 48 h after injected with TTP vectors (Scale bar=50 um) (n=6). (J) Schematic diagram of the experiment setup. Mice were first infected with either AAV-shTTP or AAV8-TTP for 2 weeks by hydrodynamic tail vein injection. Mice were then intraperitoneally injected with CCl4 for 24 h. (K) Mice were injected with AAV TTP injection for 2 weeks. The liver was collected for 1 day, 3 day, 5 day after 2 weeks injection, followed by Western blot for TTP protein. Graph shows TTP protein levels ± SEM (n=6). (L) Expression of TTP protein staining was detected in CCl4 challenged mice after AAV shCtrl or AAV TTP injection by using immunohistochemistry (n=6). (M-N) Expression of TTP protein was detected in CCl4 challenged mice after AAV shTTP injection (Scale bar=100 um). Graph shows TTP protein levels ± SEM (n=6). Data represent mean ± SEM from three independent experiments. Statistics by one-way ANOVA with Dunnett's multiple comparisons test (A ,B and K), two-way ANOVA with Tukey's multiple comparisons test (D, E, and G), two-way repeated measure ANOVA with Tukey's multiple comparisons test (H), and 2-tailed Student's t test (F, L, M, and N). *$p<0.05$ versus Ctrl.

Supplemental Figure S2

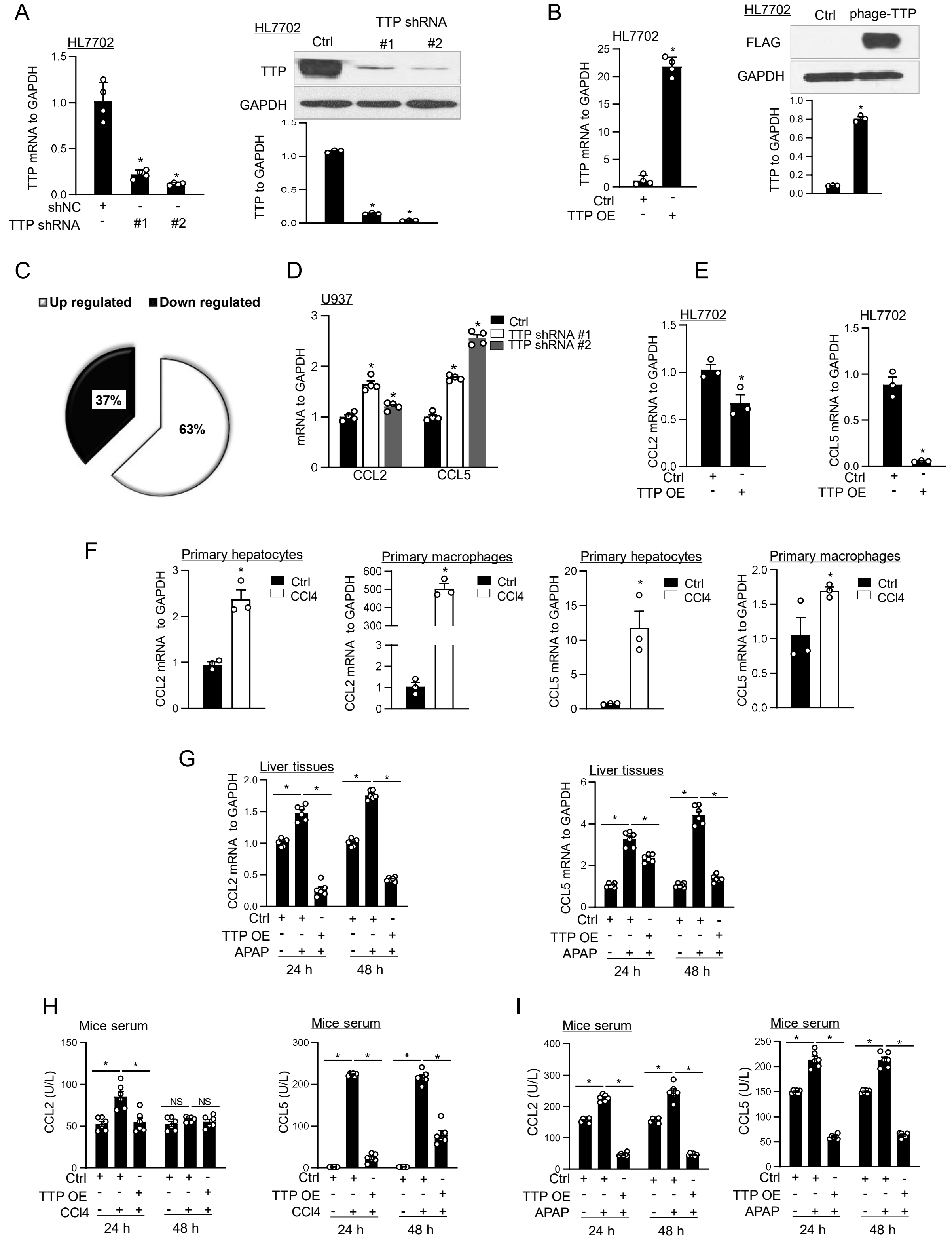

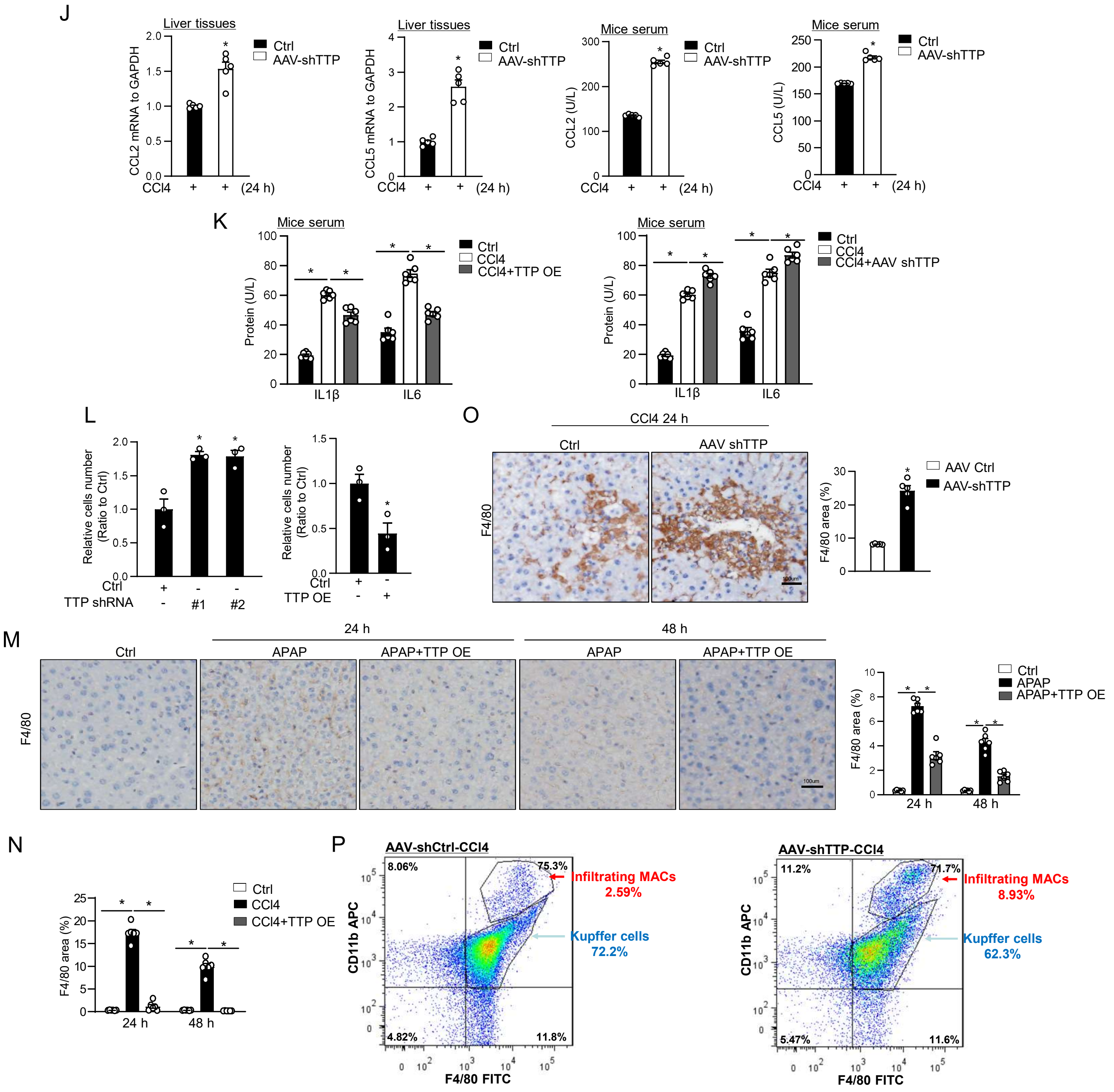

Supplemental Figure S2. TTP regulates chemokine CCL2 and CCL5 expression in vitro and in vivo. (A) The mRNA and protein level of TTP was analyzed in TTP deficiency cells by real-time PCR and Western blot. Graph shows TTP protein levels ± SEM. (B) The mRNA and protein level of TTP was analyzed in HL7702 cells transfected with Phage-TTP by real-time PCR and Western blot. Graph shows TTP protein levels ± SEM. (C) Venn diagram showing up (white)- or downregulated (black) genes in TTP knockdown cells versus Ctrl, as assessed by RNA-seq. (D-E) The mRNA level of CCL2 and CCL5 in TTP knockdown or overexpression cells was analyzed by real-time PCR. (F) The mRNA level of CCL2 and CCL5 in primary hepatocytes and macrophages isolated from CCl4 treated mice. (G) The mRNA level of CCL2 and CCL5 in APAP treated mice was analyzed by real-time PCR. (H-I) Expression of CCL2 and CCL5 proteins was measured by ELISA in mice serum from CCl4 or APAP treated mice (n=6). (J) The mRNA and protein level of CCL2 and CCL5 were analyzed in TTP deficiency mice by real-time PCR and ELISA (n=5). (K) The protein level of IL6 and IL1β in TTP deficiency or overexpression mice serum was measured by ELISA (n=6). (L) TTP knockdown or overexpression HL7702 cells were co-cultured with U937 cells. After 12 h, the number of U937 cells transmigrated into cell chambers was counted. (M) Liver sections were stained by immunohistochemistry for macrophage surface marker F4/80 to identify and quantify hepatic macrophages (Scale bar=100 um) (n=6). Graph shows F4/80 protein levels ± SEM. (N) Graph shows F4/80 protein levels ± SEM for Figure 2F. (O) F4/80 staining was conducted in TTP knockdown mice. Graph shows F4/80 protein levels ± SEM (n=6). (P) Flow cytometry was used to quantify total liver macrophages (F4/80^{+}CD11b^{+}), resident Kupffer cells (F4/80^{hi}CD11b^{lo}), as well as infiltrating liver macrophages (F4/80^{lo}CD11b^{hi}) (n=6). Data represent mean ± SEM from three independent experiments. Statistics by one-way ANOVA with Dunnett's multiple comparisons test (A, D, and L), 2-tailed Student's t test (B, E, F, J, and O), and two-way ANOVA with Tukey's multiple comparisons test (G, H, I, K, M, and N). *p<0.05 versus Ctrl.

Supplemental Figure S3

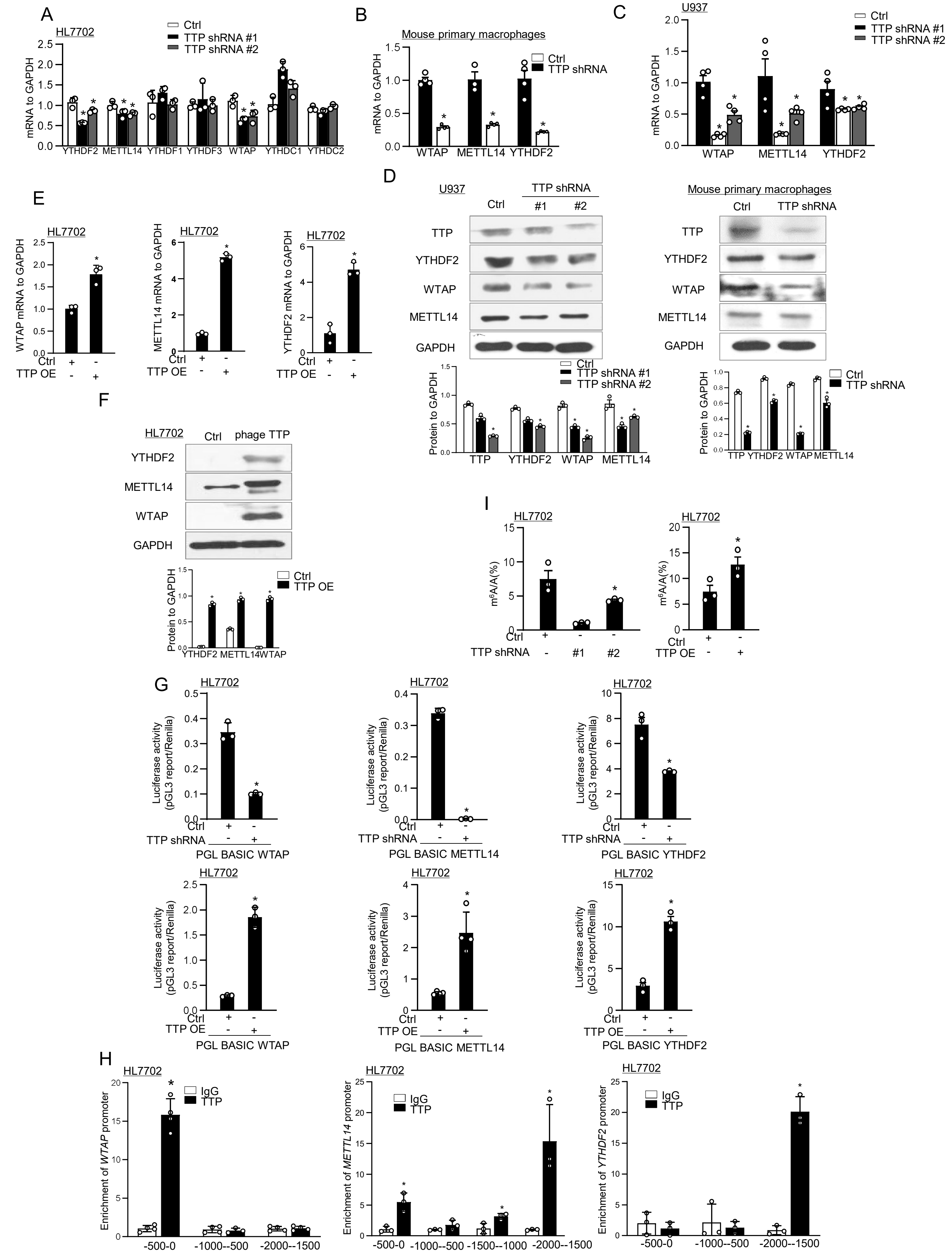

Supplemental Figure S3. TTP actives the expression of several m^{6}A-associated genes. (A) m^{6}A associated genes were analyzed in TTP knockdown cells by real-time PCR. (B) Real-time PCR for WTAP, METTL14, and YTHDF2 mRNA levels in mouse primary macrophages transfected with shTTP. (C) Real-time PCR for WTAP, METTL14 and YTHDF2 mRNA levels in U937 cells transfected with shTTP. (D) Western blot for WTAP, METTL14, and YTHDF2 protein in TTP knockdown cells. Graph shows protein levels $\pm$ SEM. (E) Real-time PCR for WTAP, METTL14, and YTHDF2 mRNA levels in HL7702 cells transfected with phage-TTP. (F) Western blot for WTAP, METTL14, and YTHDF2 protein in TTP overexpression cells. Graph shows protein levels $\pm$ SEM. (G) Stable TTP knockdown or overexpression HL7702 cells were transfected with the promoters of *WTAP*, *METTL14*, or *YTHDF2* for 48 h, followed by luciferase analysis. (H) ChIP analysis of the recruitment of TTP to *WTAP*, *METTL14*, and *YTHDF2* promoter regions in HL7702 cells. (I) The m^{6}A levels of total RNAs in TTP knockdown or overexpression cells. Data represent mean $\pm$ SEM from three independent experiments. Statistics by one-way ANOVA with Dunnett's multiple comparisons test (A, C, D, and I), 2-tailed Student's t test (B, E, F, G, and H). *p<0.05 versus Ctrl.

Supplemental Figure S4

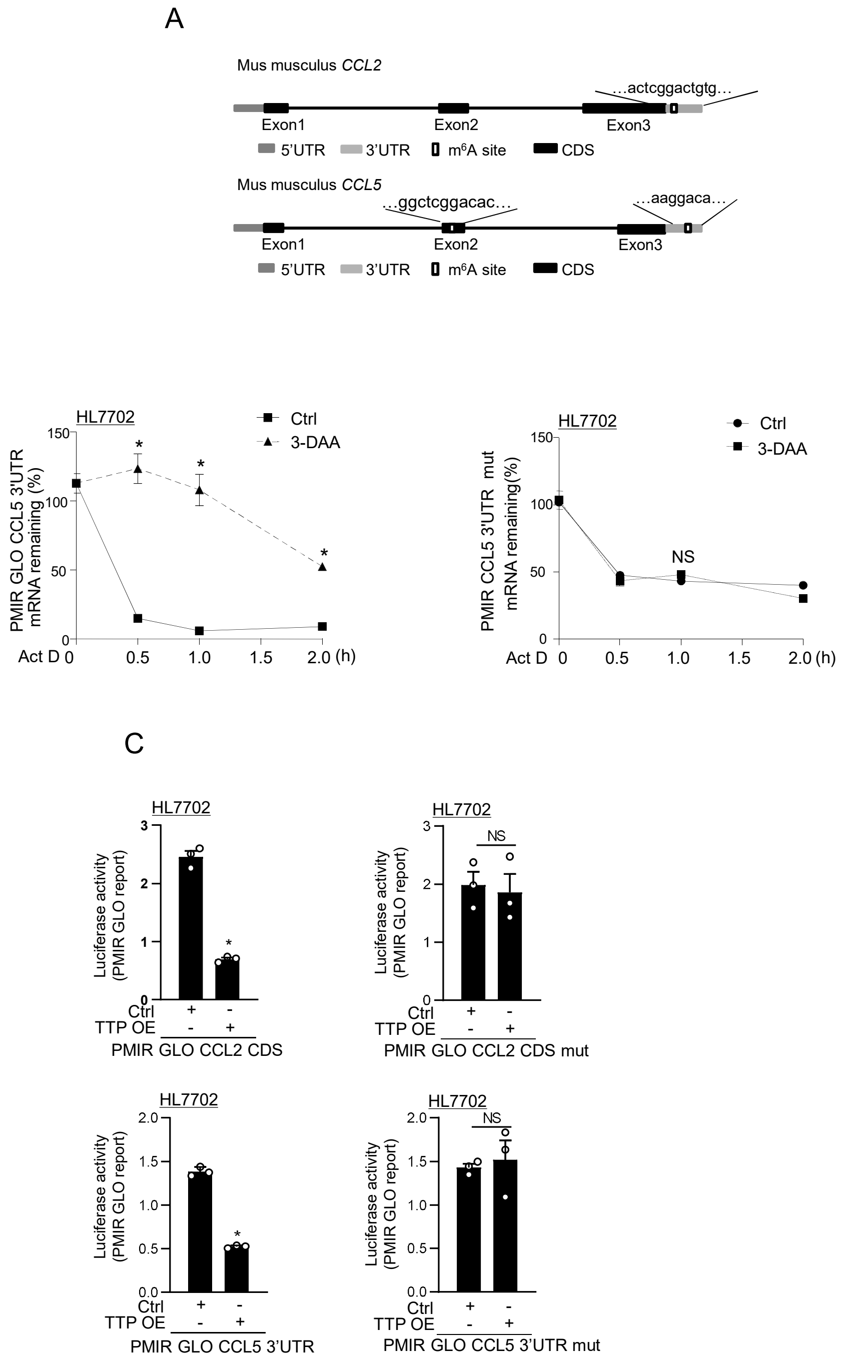

Supplemental Figure S4. TTP regulates the stabilization of CCL5 mRNAs through m^{6}A modification. (A) Schematic structures showing the potential m^{6}A sequences on Mus musculus *Ccl2* and *Ccl5* mRNAs. (B) The stability of PMIR-GLO-CCL5-3'UTR or mutated vectors mRNAs in HL7702 cells with or without 3-DAA treatment. (C) Stable TTP overexpression cells were transfected with of PMIR-GLO-CCL2-CDS, PMIR-GLO-CCL2-CDS mut, PMIR-GLO-CCL5-3′UTR, or PMIR-GLO-CCL5-3'UTR mut for 48 h, followed by luciferase analysis. Data represent mean ± SEM from three independent experiments. Statistics by 2-tailed Student's t test. *p<0.05 versus Ctrl. NS means no significant difference versus Ctrl.

Supplemental Figure S5

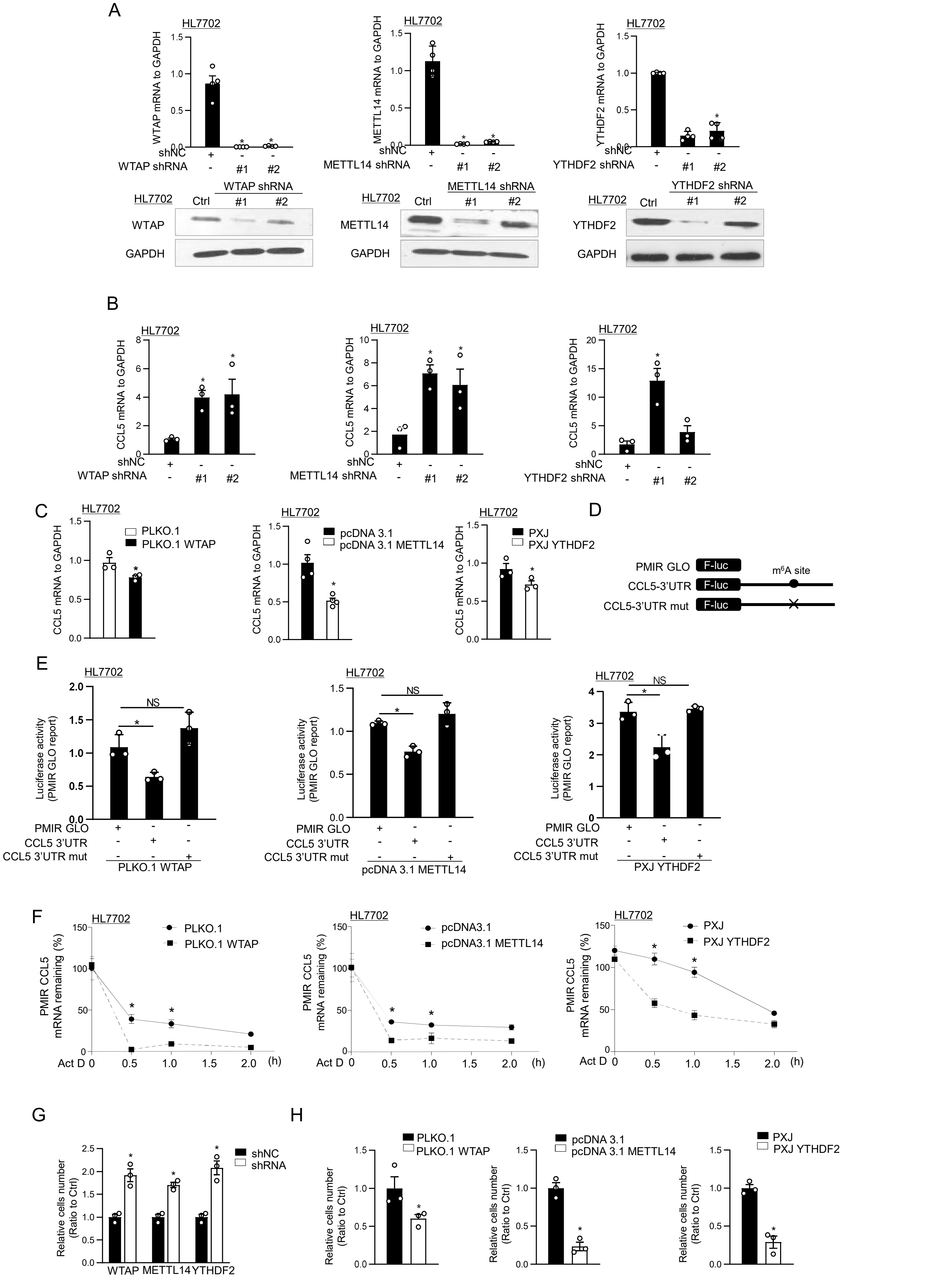

Supplemental Figure S5. WTAP, METTL14, and YTHDF2 regulates the stabilization of CCL5 mRNA by targeting m^{6}A sites. (A) The mRNA and protein level of WTAP, METTL14, and YTHDF2 in HL7702 cells stably transfected with WTAP, METTL14, or YTHDF2 shRNA were analyzed by real-time PCR and Western blot (B) The effects of WTAP, METTL14 and YTHDF2 knockdown on expression levels of CCL5 in HL7702 cells. (C) Effects of WTAP, METTL14 and YTHDF2 overexpression on CCL5 expression levels in HL7702 cells. (D) Schematic representation of mutation in m^{6}A sites. (E) HL7702 cells were co-transfected with the luciferase construct containing the 3'UTR of CCL5 (PMIR-GLO-CCL5-3'UTR) or CCL5 3'UTR with mutation (PMIR-GLO-CCL5-3'UTR-mut) and WTAP, METTL14, or YTHDF2 overexpression plasmid for 48 h, followed by luciferase analysis. (F) The stability of CCL5 mRNA was calculated in HL7702 cells transfected with WTAP, METTL14, or YTHDF2 overexpression plasmid. (G-H) HL7702 cells were stably transfected with WTAP, METTL14, or YTHDF2 shRNA /overexpression vector for 48 h. HL7702 cells were then cocultured with U937 cells. After 12 h, the number of U937 cells transmigrated into cell chambers was counted. Data represent mean ±SEM from three independent experiments. Statistics by one-way ANOVA with Dunnett's multiple comparisons test (A, B, and E), 2-tailed Student's t test (C, F, G, and H). *$p<0.05$ versus Ctrl. NS means no significant difference versus Ctrl.

Supplemental Figure S6

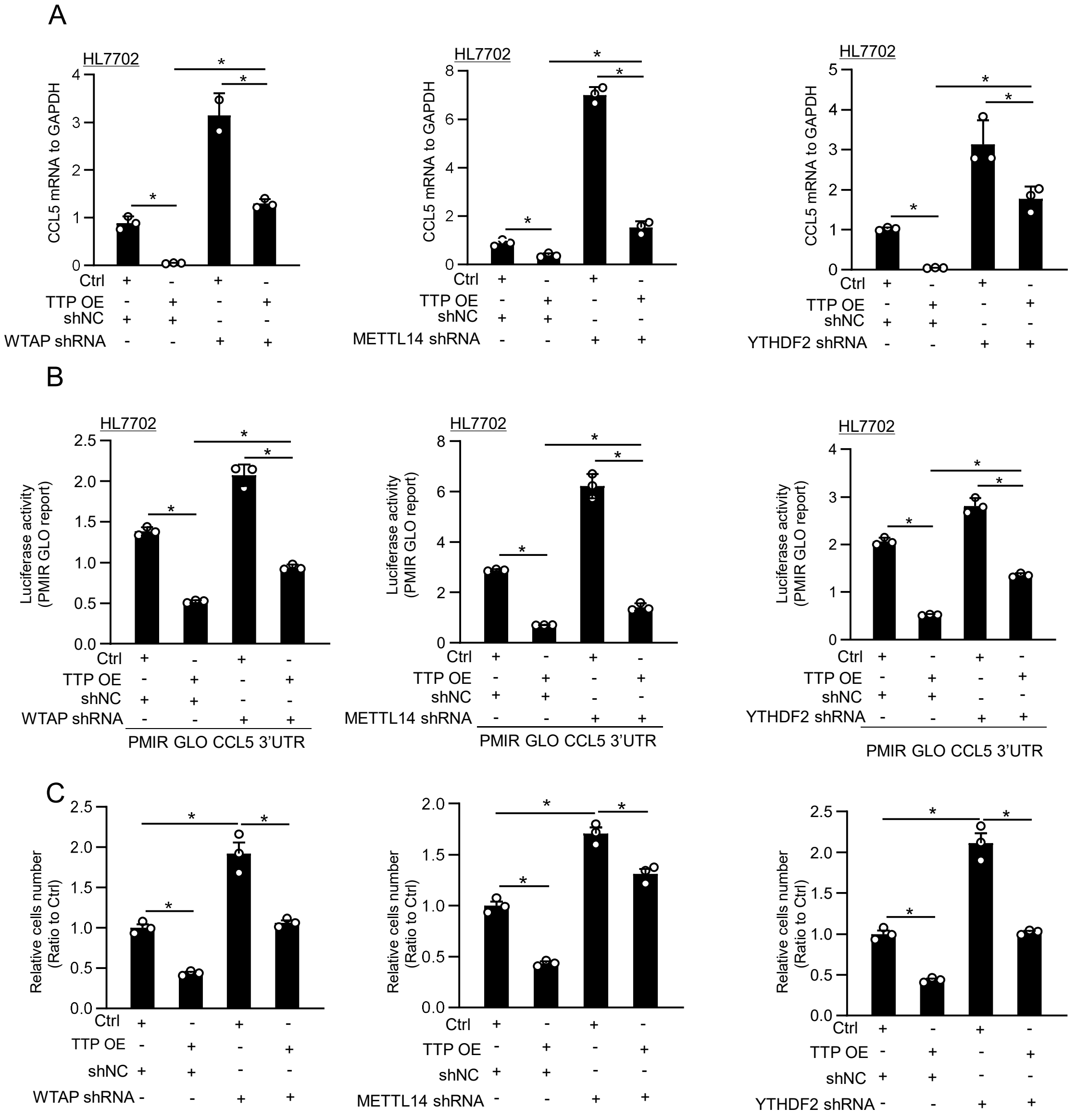

Supplemental Figure S6. TTP affects m^{6}A-mediated post-transcription of CCL5 though regulation of WTAP, METTL14, and YTHDF2. (A) The level of CCL5 was analyzed in WTAP, METTL14, or YTHDF2 knockdown cells transfected with TTP overexpression vector by real-time PCR . (B) HL7702 cells were co-transfected with the luciferase construct containing 3'UTR of CCL5 (PMIR-GLO-CCL5- 3'UTR) and WTAP, METTL14 or YTHDF2 shRNA and TTP overexpression vector for 48 h, followed by luciferase analysis. (C) HL7702 cells were co-transfected with WTAP, METTL14 or YTHDF2 shRNA and TTP overexpression vector, and then cocultured with U937 cells. After 12 h, the number of U937 cells transmigrated into cell chambers was counted. Data represent mean ± SEM from three independent experiments. Statistics by two-way ANOVA with Tukey's multiple comparisons test. *p<0.05 versus Ctrl.

Supplemental Figure S7

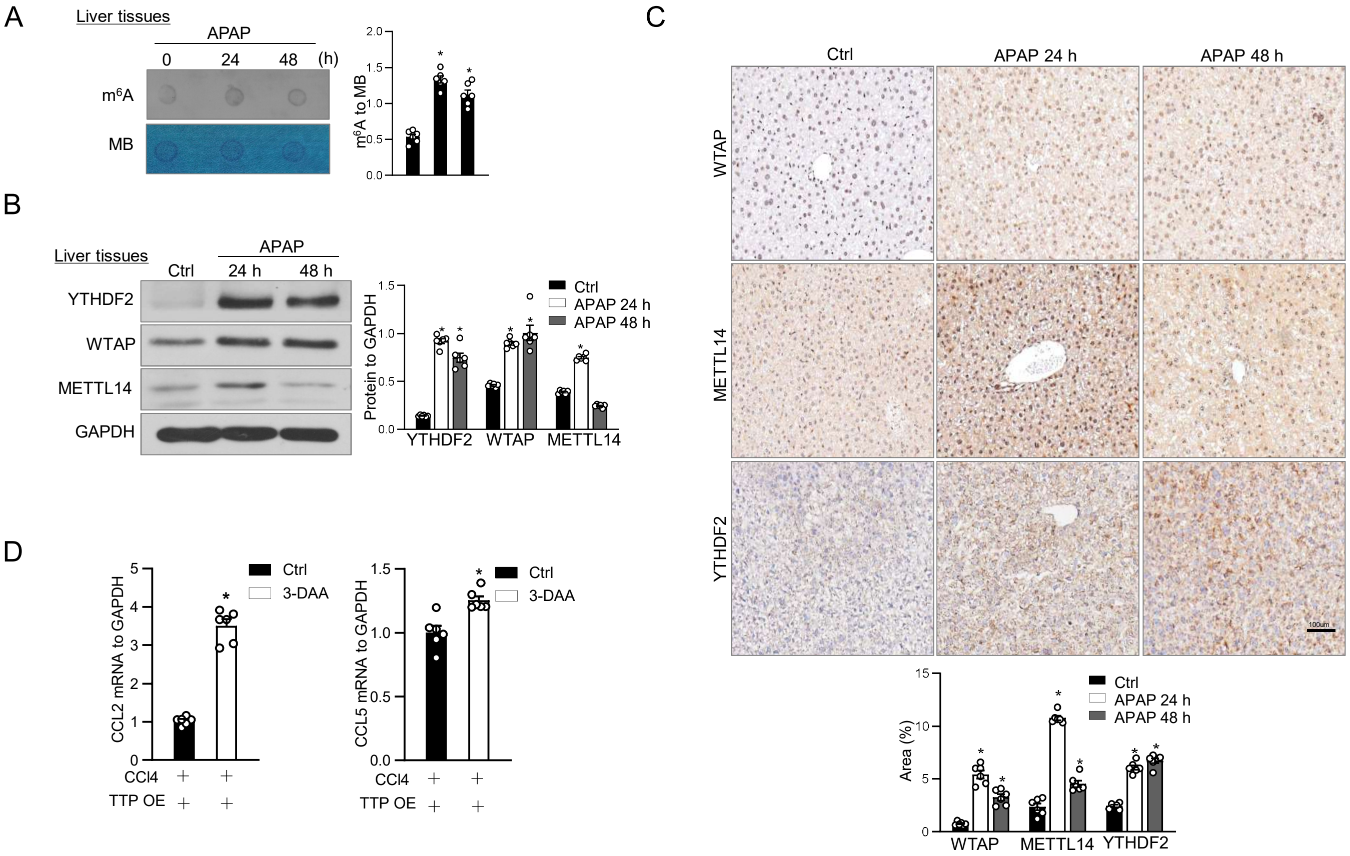

Supplemental Figure S7. m^{6}A methylation is involved in TTP-mediated protection against ALF. (A) Dot blot was used to measure m^{6}A modification in the mice livers induced by APAP for different time. (B) The protein level of WTAP, METTL14, and YTHDF2 was detected in mice induced by APAP for different time. Graph shows protein levels ± SEM (n=6). (C) Expression of WTAP, METTL14, and YTHDF2 protein staining was detected in the liver from mice induced by APAP for different time (Scale bar=100 um) (n=6). Graph shows protein levels ± SEM. (D) The mRNA level of CCL2 and CCL5 in CCl4 challenged mice treated with 3-DAA was measured by real-time PCR. Data represent mean ± SEM from three independent experiments. Statistics by one-way ANOVA with Dunnett's multiple comparisons test (A, B, and C) and) and 2-tailed Student's t test (D). *p<0.05 versus Ctrl.

Supplemental Table S1. Primers and shRNA sequences

Gene symbol	Sense primer (5'-3')	Antisense primer (5'-3')
Primers for real-time PCR		
TTP	TCGGGACCCTGGAGCCTGAG	AGCCAGCGGTGCGAAGCC
WTAP	TGGCGAAGTGTCGAATGCTTA	CAACTGCTGGCGTGTCTCCTT
METTL14	TGGACTTGGGATGATATTATGA	CCCATTTTCGTAAACACACTCT
YTHDF2	TAGCCAACTGCGACACATTC	CACGACCTTGACGTTCCTTT
CCL2	AATCACCAGCAGCAAGTGTCCC	AAGTCTTCGGAGTTTGGGTTTGC
CCL5	CCTCGCTGTCATCCTCATTGCTAC	CAGTGGGCGGGCAATGTAGG
luciferase	CACCGTCGTATTCGTGAGCAA	CGGTGGCAAATGGGAAGTCA
GAPDH	ACCATCTTCCAGGAGCGAGATC	TGATGACCCTTTTGGCTCCCC
m-GAPDH	ATGGTGAAGGTCGGTGTGAA	CGCTCCTGGAGATGGTGAT
m-Ccl2	GTGCTGACCCCAAGAAGGAATG	AGTGCTTGAGGTGGTTGTGGAAA
m-Ccl5	GGACACCACTCCCTGCTGCTTT	CACTTCTTCTCTGGGTTGGCACAC
Sequences for shRNAs		
Ctrl	TTCTCCGAACGTGTCACGTT	
TTP shRNA 1	CCAGAGCATCAGCTTCTCGAGAAGCT	
TTP shRNA 2	TACAAGACTGAGCTATGTCTCGAGACA	
METTL14 shRNA 1	CCATGTACTTACAAGCCGATA	
METTL14 shRNA 2	GCTTACAAATAGCAACTACAA	
WTAP shRNA 1	CAAGAGATGAGTTCTCGAGAATTAAC	
WTAP shRNA 2	TGGCAAGAGATGAGTTACTCGAGTAAC	
YTHDF2 shRNA 1	TCTGGATATAGTAGCAATTAT	
YTHDF2 shRNA 2	CACTTCTTCTCTGGGTTGGCACAC	
m-TTP shRNA	ACCACCTCCTCTCGATACAAGCTCGAGC	
Primers for RIP-PCR / MeRIP-PCR/ iCLIP-PCR		
CCL2 CDS contains m^6A site	ATCACCAGCAGCAAGTGTCC	AAGTCTTCGGAGTTTGGGTTT
CCL2 contains ARE	ACACTCACTCCACAACCCAAG	GTGGTTCAAGAGGAAAAGCAAT
CCL5 3'UTR contains m^6A site	CCAGATTCTACCACACAGCAGCAGT	TTCACGCCATTCTCCTGCCTC
CCL5 CDS contains m^6A site	ATCACCAGCAGCAAGTGTCC	AAGTCTTCGGAGTTTGGGTTT
Primers for ChIP-PCR		
WTAP promoter (-2000- -1500)	AAAACAGAAGTACAGTGGGATG	ATGCAGGAGATTGGATTTAG
WTAP promoter (-1000- -500)	CATTCCCAATCAGTTCCCTAT	TCAAGTTCCACGGAGTACAAA
WTAP promoter (-500- 0)	TCCAGACCGATCTGATTCACTG	GCTTTATCAAACCTGTTCATCCC
METTL14 promoter (-2000- -1500)	TGTAAGGGACATAGTCATCG	TTGCTTGTAATCTTTGAACCC
METTL14 promoter (-1500- -1000)	TATAGACTCTAGGGCCTATCA	CAGTTGTATTATCTGGCAATC
METTL14 promoter (-1000- -500)	CGCAATCCGAGTAATTTCAGG	AAGCGTGTCTTGGTTTATGTCCT
METTL14 promoter (-500- 0)	GACAAAACTGAGGCTCACGAA	CCAGGATAGCAGGTTCCCTT
YTHDF2 promoter (-2000- -1500)	GAAACCATTATTTAGACCCTTTGTC	GCGGTTGCCTATACTTACCCT
YTHDF2 promoter (-1000- -500)	AGTTGGCTTTGCTTCTGATTG	CCAGGCTTTGTTGTTAGGTATT
YTHDF2 promoter (-500- 0)	CGGCTAACAGAGGATGACCAA	TCTGGCTTCAGAAGAACGACAA
Primers for plasmid construction		
PGL *WTAP*	actcgtTTGCTTGAGCCCAGGAAGTCA	aagcttGTGGAGTAGACTCCCTGAAGTAAGA
PGL *METTL14*	gagctcGACGCCATTCACTATGGAGAAATA	aagcttACTTCTCCTTTATCTCTCTTTCCCA
PGL *YTHDF2*	acgcgtAACTGGGAACTTCTGGCAAAC	ctcgagTAAAGCAGAGCGCCGAACA

Restriction enzyme sites were indicated by lowercase letters
